# Interactions of Bacterial Toxin CNF1 and Host JAK1/2 Driven by Liquid-Liquid Phase Separation Enhance Macrophage Polarization

**DOI:** 10.1128/mbio.01147-22

**Published:** 2022-06-29

**Authors:** Xuan Sun, Jianming Yang, Xueqin Deng, Yuting Wei, Changying Wang, Yaxiu Guo, Huan Yang, Liu Yang, Chunhui Miao, Junqiang Lv, Yawen Xiao, Hong Zhang, Zhi Yao, Quan Wang

**Affiliations:** a Key Laboratory of Immune Microenvironment and Disease (Ministry of Education), Tianjin Institute of Urology, Department of Immunology, School of Basic Medical Sciences, Tianjin Medical University, Tianjin, China; b The Province and Ministry Co-sponsored Collaborative Innovation Center for Medical Epigenetics, Tianjin Medical University, Tianjin, China; Texas & Health Science Center; Cornell University

**Keywords:** CNF1, JAK-STAT1, liquid-liquid phase separation, macrophage polarization

## Abstract

Urinary tract infections (UTIs) are a global public health concern, which is mainly caused by uropathogenic Escherichia coli (UPEC). Cytotoxic necrotizing factor 1 (CNF1) is a key UPEC toxin and regulates multiple host cellular processes through activating the Rho GTPases; however, the effect of CNF1 on macrophage polarization remains unknown. Here, we found that CNF1 promoted M1 macrophage polarization through regulating NF-κB and JAK-STAT1 signaling pathways in kidney at an early stage of acute UTIs. Notably, we identified CNF1 could directly interact with JAK1/2 through its domain without Rho GTPases activation, which induced JAK1/2 phosphorylation, subsequent STAT1 activation and M1 polarization. Moreover, CNF1 exhibited liquid-liquid phase separation (LLPS) to induce a CNF1-JAK1/2 complex, promoting macrophage reprogramming. These findings highlight the LLPS-dependent and Rho GTPase-independent effect of CNF1 as an adaptor on interfering with host cell signals.

## INTRODUCTION

Urinary tract infections (UTIs) are identified as one of the most common bacterial infections and are a global public health concern (1, 2). Uropathogenic Escherichia coli (UPEC) is the main etiological factor of UTIs, and its pathogenicity is dependent on its virulence factors (3–6). UTIs caused by UPEC can lead to pyelonephritis, cystitis, prostatitis, and bacteremia (7).

Cytotoxic necrotizing factor 1 (CNF1) is a key toxin secreted by UPEC, which can be taken up into host cells by receptor-mediated endocytosis (8). In the receptor-mediated endocytosis, CNF1 binds to 67-kDa laminin receptor (67LR) and 37-kDa laminin receptor precursor (p37/LRP) through the N terminus (amino acids 1 to 342) and to Lutheran adhesion glycoprotein/basal cell adhesion molecule (Lu/BCAM) through the region (amino acids 709 to 730). After binding to its receptor, CNF1 enters endocytic vesicles by receptor-mediated endocytosis and is subsequently transferred to an endosomal compartment. In the late endosome, CNF1 inserts into membranes at acidic pH to drive the translocation of the catalytic domain into the cytosol through two hydrophobic alpha-helices located in the membrane translocation domain. After the translocation, an approximately 55-kDa fragment containing the catalytic domain and an additional part (amino acids 542 to 1014) is cleaved off and released from the endosomal membrane into the cytosol (9). After translocation into cytosol, the enzymatic domain of CNF1 induces posttranslational deamidation on several Rho GTPases, resulting in their activation (10). A recent study demonstrates that CNF1 induces NLRP3 inflammasome activation through the Rac2-Pak-NLRP3 axis (11). We have reported that CNF1 accelerates prostate cancer progression through activating the Cdc42-PAK1 axis, induces vascular endothelial growth factor (VEGF) and angiogenesis through the RhoC-HSF1-HSP90α-HIF1α axis, and downregulates CD36 mediated phagocytosis through the Cdc42-LXRβ axis (8, 12, 13). Based on these published studies, CNF1 pathways depending on its Rho GTPases activation; however, its Rho GTPase-independent role in interfering with host cell signals is unknown.

Macrophages, the key cells in innate immune response, play an important role in host defenses against UPEC during UTIs (14, 15). Tissue-resident sentinel macrophages sensed UPEC during UTIs and produced chemokines to recruit neutrophils and blood monocytes into the infected uroepithelium. Recent studies also showed that macrophages directly phagocytose UPEC and retain free iron to limit UPEC growth to reduce infection (16–18). Through macrophages contribute to bacterial clearance, its excessive amount result in exacerbated inflammation and tissue damage (19). We previously reported that alpha-hemolysin (HlyA) of UPEC induced macrophage accumulation to enhance kidney injury (20). Macrophages are usually classified into two phenotypes including the classical activation (M1) and alternative activation (M2) type, and the imbalance of M1 and M2 polarization affect inflammatory responses (21). M1 macrophages express tumor necrosis factor alpha (TNF-α), interleukin-6 (IL-6), IL-12, IL-1β, and inducible nitric oxide synthase contributing to proinflammatory and antimicrobial functions, whereas M2 macrophages express Tgf-β, Ym1, Mrc1, Pparγ, and IL-10 promoting anti-inflammation and tissue repair (22). Janus kinase-signal transducers and activators of transcription (JAK-STAT), nuclear factor-kappa B (NF-κB) and mitogen-activated protein kinase (MAPK) pathways are involved in macrophage polarization (23, 24). During bacterial infections, macrophages typically exhibit an M1-like phenotype and restrict pathogens through cytokine and chemokine at an early stage (25). However, uncontrolled M1 macrophage increasement induces severe inflammation such as gastroenteritis, pyelonephritis, neonatal meningitis, and sepsis (26). We have previous found that CNF1 induces urinary tract inflammation and reduces macrophage phagocytosis of UPEC (13); however, the effect of CNF1 on macrophage polarization has not been reported.

Liquid-liquid phase separation (LLPS) is considered the underlying driving force for membraneless compartmentalization in cells (27). Growing evidence suggests that LLPS regulates various physiological processes such as enzymatic reactions and signal transduction (28). A bacterial effector, XopR, has recently been reported to undergo LLPS, which hijacks and subverts the host *Arabidopsis* actin cytoskeleton (29). Whether CNF1 exhibits the LLPS phenomenon is unknown.

In this study, we found that CNF1 promoted M1 macrophage polarization through regulating NF-κB and JAK1/2-STAT1 signaling pathways in kidney at an early stage of acute UTIs. Notably, we demonstrated that the CNF1 domain without Rho GTPases activation physically bound to JAK1 and JAK2 to form a protein complex through LLPS, which induced JAK1/2 phosphorylation and the following STAT1 activation. Our results highlight the LLPS-dependent and Rho GTPase-independent effect of CNF1 on interfering with cell signals to induce macrophage reprogramming.

## RESULTS

### CNF1 promotes M1 macrophage polarization in kidney using an acute pyelonephritis mouse model.

**FIG 1:**
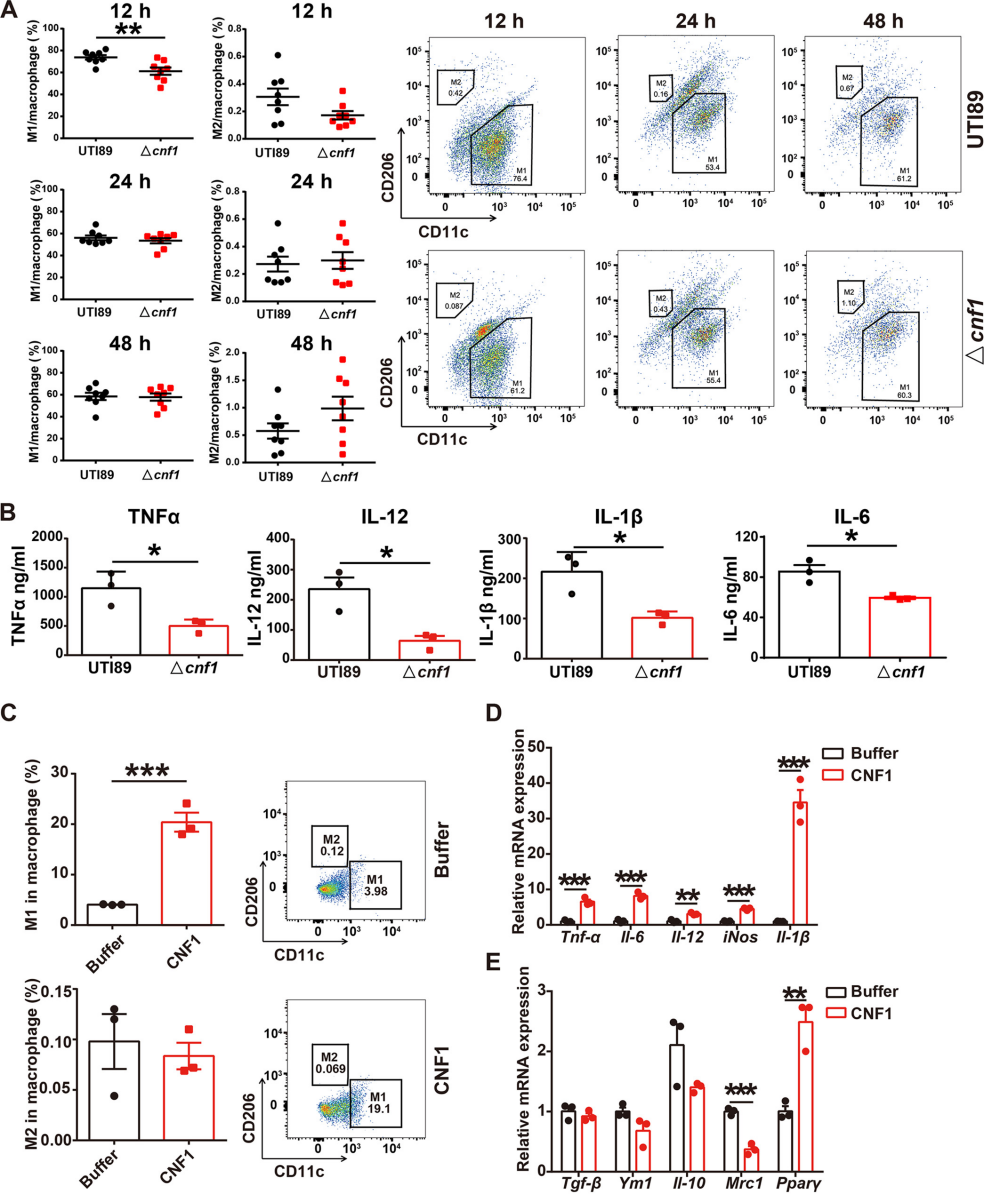
CNF1 induces M1 macrophage polarization *in vivo* and *in vitro*. (A and B) Female C57BL/6J mice were inoculated intraurethrally with 10^8^ CFU of the UTI89 or Δ*cnf1* strain two times at 3-h intervals, respectively. (A) Flow cytometry analysis showing the percentage of M1 and M2 macrophages in kidney cells at 12, 24, and 48 hpi (left) (*n* = 8). Representative flow cytometry dot plots are shown (right). (B) ELISA results showing TNF-α, IL-12, IL-1β, and IL-6 levels in kidney tissues at 12 hpi (*n* = 3). (C to E) BMDMs were treated with CNF1 (3 nM) and dialysis buffer for 6 h (*n* = 3). (C) Flow cytometry results showing the percentages of M1 and M2 macrophages (left). Representative flow cytometry dot plots are shown (right). (D) mRNA levels of M1-characterized markers *Tnf-α*, *Il-6*, *Il-12*, *iNos*, and *Il-1β*. (E) mRNA levels of M2-characterized markers *Tgf-β*, *Ym1*, *Il-10*, *Mrc1*, and *Pparγ*. The data represent means ± the SEM. A nonparametric Mann-Whitney test (A) or Student *t* test (B to E) was performed (*, *P* < 0.05; **, *P* < 0.01; ***, *P* < 0.001).

To examine whether CNF1 affects macrophage polarization in kidney, UPEC strains UTI89 and a *cnf1* deletion strain derived from UTI89 (Δ*cnf1*) were used to transurethrally infect female C57BL/6J mice. M1 and M2 macrophages in kidney tissues of mice infected with UTI89 and Δ*cnf1* strains at 12, 24, or 48 h postinfection (hpi) were analyzed using flow cytometry. Significantly increased percentages and numbers of M1 macrophages were detected in the UTI89 group at 12 hpi compared to those in the Δ*cnf1* group, while no difference was observed at 24 or 48 hpi (Fig. 1A; see also Fig. S1A and B in the supplemental material). We also examined M1 and M2 macrophages in kidneys of mice transurethrally given equal amounts of phosphate-buffered saline (PBS) and found that the percentages and numbers of M2 macrophages were higher and the percentages and numbers of M1 macrophages were lower compared to those in infected mice (Fig. 1A; see also Fig. S1A and B). Notably, the percentage and number of M2 macrophages were similar between the UTI89 and Δ*cnf1* group (Fig. 1A; see also Fig. S1A and B). To rule out the possibility that low bacterial burdens elicited less macrophage polarization in the Δ*cnf1* group, we further determined the bacterial burdens at 6 and 12 hpi in kidney tissues. The results showed that bacterial burdens at 6 and 12 hpi in kidney tissues were similar between the UTI89 and Δ*cnf1* groups (see Fig. S1C). Moreover, the levels of M1 signature proinflammatory cytokines TNF-α, IL-6, IL-12, and IL-1β were increased in kidney tissues of UTI89 group compared to those of the Δ*cnf1* group as determined by enzyme-linked immunosorbent assay (ELISA) (Fig. 1B). These results indicate that CNF1 promotes M1 macrophage polarization in kidney at an early stage of acute pyelonephritis.

10.1128/mbio.01147-22.1 FIG S1 Effects of CNF1 on M2 macrophage polarization *in vivo* and *in vitro*. (A) Gating strategy for flow cytometry analysis of M1 and M2 macrophages. (B) Female C57BL/6J mice were inoculated intraurethrally with PBS or 10^8^ CFU of the UTI89 or Δ*cnf1* strain two times at 3-h intervals, respectively. The numbers of M1 and M2 macrophages in kidney cells were analyzed at 12, 24, and 48 hpi for mice infected with the UTI89 or Δ*cnf1* strain and at 12 hpi for mice injected with PBS (*n* = 8). (C) Female C57BL/6J mice were inoculated intraurethrally with 10^8^ CFU of the UTI89 or Δ*cnf1* strain two times at 3-h intervals. Bacterial titers in the kidney were assessed at 6 and 12 hpi, respectively (*n* = 8). (D and E) BMDMs were infected with the UTI89 or Δ*cnf1* strain for 6 h at an MOI of 5 (*n* = 3). (D) Flow cytometry analysis showing the percentages of M1 and M2 macrophages. (E) mRNA levels of M1-characterized markers *Tnf-α*, *Il-6*, *Il-12*, *iNos*, and *Il-1β* (left) and mRNA levels of M2-characterized markers *Tgf-β*, *Ym1*, *Il-10*, *Mrc1*, and *Pparγ* (right). (F to H) Effect of ERK inhibitor (SCH772984), p38 inhibitor (SB 239063), or JNK inhibitor (JNK-IN-8) on mRNA levels of *Tnf-α*, *Il-6*, *Il-12*, *iNos*, and *Il-1β* in the indicated BMDMs (*n* = 3). (I and J) Western blotting of pSTAT3 (Ser727)/STAT3 and pSTAT5 (Tyr694)/STAT5 protein levels in the indicated BMDMs and THP-1 cells. (K to M) Representative flow cytometry dot plots of M1 and M2 macrophages in the indicated BMDMs treated with NF-κB inhibitor (Bay 11-7085), JAK1/2 inhibitor (AZD1480), or STAT1 inhibitor (Fludarabine). (N) Western blot showing the effect of NF-κB inhibitor (Bay 11-7085) on pSTAT1 (Tyr701)/STAT1 in the indicated BMDMs. (O) Western blot showing the effect of STAT1 inhibitor (Fludarabine) on pIκBα (Ser32)/IκBα in indicated BMDMs. (P and Q) Western blot showing the effect of JAK1/2 inhibitor (AZD1480) on pIκBα (Ser32)/IκBα and pSTAT1 (Tyr701)/STAT1 in the indicated BMDMs. The data represent the means ± the SEM. A nonparametric Mann-Whitney test (B and C), Student *t* test (D and E), or one-way ANOVA (F to H) was performed (*, *P* < 0.05; **, *P* < 0.01; ***, *P* < 0.001; ****, *P* < 0.0001). Download FIG S1, TIF file, 2.9 MB. Copyright © 2022 Sun et al. 2022 Sun et al. https://creativecommons.org/licenses/by/4.0/ This content is distributed under the terms of the Creative Commons Attribution 4.0 International license.

### CNF1 induces BMDMs toward M1 macrophage polarization *in vitro*.

Since CNF1 seemed to play a role in M1 macrophage polarization *in vivo*, we sought to determine whether CNF1 drives M1 macrophage polarization *in vitro*. Bone marrow-derived macrophages (BMDMs) were infected with UTI89 and Δ*cnf1* bacteria, and the percentages of M1 macrophages were ~2-fold increased in BMDMs infected with UTI89 but not for M2 macrophages (see Fig. S1D and Fig. S1A). Accordingly, the mRNA levels of *Tnf-α*, *Il-6*, *Il-12*, *iNos*, and *Il-1β* were dramatically increased in BMDMs infected with UTI89, while the mRNA levels of most M2-characterized markers, including *Tgf-β*, *Pparγ*, *Mrc1*, and *Ym1*, were not increased (see Fig. S1E). Next, we treated BMDMs with recombinant CNF1 or dialysis buffer for 6 h and examined the percentages of M1 macrophages by flow cytometry. The percentages of M1 macrophages were significantly increased in the CNF1 group (Fig. 1C; see also Fig. S1A). Moreover, the mRNA levels of *Tnf-α*, *Il-6*, *Il-12*, *iNos*, and *Il-1β* were significantly increased in BMDMs treated with CNF1 (Fig. 1D), while the mRNA levels of the M2-characterized markers *Tgf-β*, *Ym1*, *Il-10*, and *Mrc1*, but not *Pparγ*, were not increased in the CNF1 group (Fig. 1E). These results suggest that CNF1 directly induces M1 macrophage polarization.

### CNF1 impacts macrophage polarization through the NF-κB and JAK-STAT1 signaling pathways.

**FIG 2:**
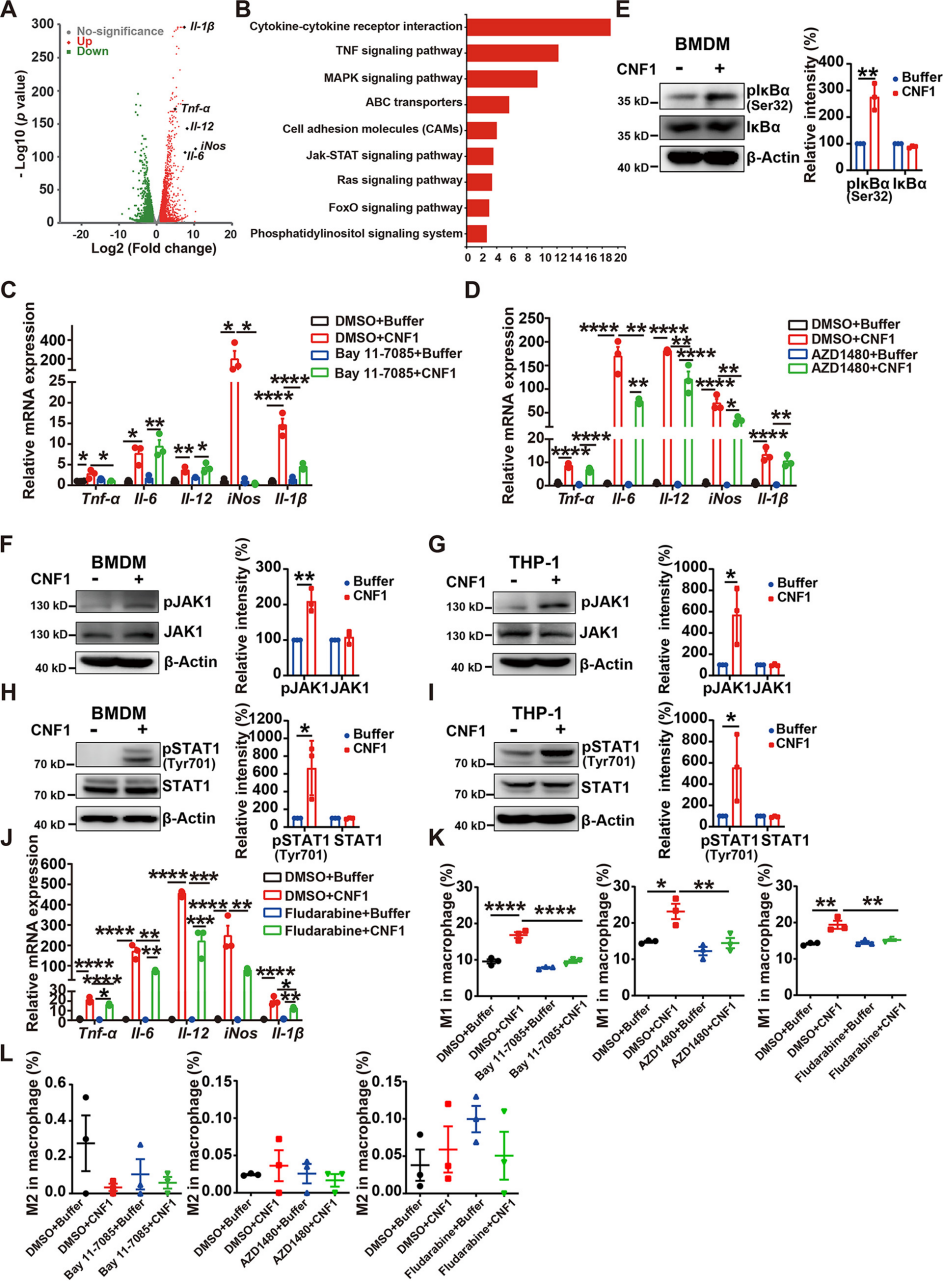
CNF1 impacts M1 macrophage polarization through NF-κB and JAK-STAT1 signaling pathways. (A and B) RNA-seq analysis of BMDMs treated with CNF1 (3 nM) or dialysis buffer for 6 h (*n* = 3). (A) Volcano plots show the upregulated and downregulated genes in CNF1-treated BMDMs compared to dialysis buffer-treated BMDMs. Green indicates downregulated genes, red indicates upregulated genes, and gray indicates nondifferentially expressed genes. (B) KEGG analysis showing the upregulated pathways in CNF1-treated BMDMs compared to dialysis buffer-treated BMDMs. (C and D) Effects of NF-κB inhibitor (Bay 11-7085) (C) and JAK1/2 inhibitor (AZD1480) (D) on mRNA levels of *Tnf-α*, *Il-6*, *Il-12*, *iNos*, and *Il-1β* in the indicated BMDMs (*n* = 3). (E) Western blot of pIκBα/IκBα protein levels in the indicated BMDMs. The signal densities of pIκBα were normalized to that of β-actin and total IκBα. The signal densities of IκBα were normalized to that of β-actin. The relative density of buffer-treated cells was set to 100%. (F to I) Western blots of pJAK1/JAK1 (F and G) and pSTAT1 (Tyr701)/STAT1 (H-I) protein levels in the indicated BMDMs and THP-1 cells. The signal densities of pJAK1 were normalized to that of β-actin and total JAK1. The signal densities of pSTAT1 were normalized to that of β-actin and total STAT1. The signal densities of total JAK1 and STAT1 were normalized to that of β-actin. The relative density of buffer-treated cells was set to 100%. (J) Effect of STAT1 inhibitor (Fludarabine) on mRNA levels of *Tnf-α*, *Il-6*, *Il-12*, *iNos*, and *Il-1β* in the indicated BMDMs (*n* = 3). (K to L) Flow cytometry analysis results showing the effect of NF-κB inhibitor (Bay 11-7085), JAK1/2 inhibitor (AZD1480), or STAT1 inhibitor (Fludarabine) on the percentages of M1 macrophages (K) and M2 macrophages (L) in the indicated BMDMs (*n* = 3). The data represent the means ± the SEM. One-way ANOVA (C, D, J, and L) or two-way ANOVA (E to I) were performed (*, *P* < 0.05; **, *P* < 0.01; ***, *P* < 0.001; ****, *P* < 0.0001).

To study the molecular mechanisms through which CNF1 induces M1 macrophage polarization, we analyzed gene expression profiles of BMDMs treated with CNF1 or dialysis buffer. A total of 2,062 genes, including the M1-characterized markers *Tnf-α*, *Il-6*, *Il-12*, *iNos*, and *Il-1β*, were upregulated, and 1,824 genes were downregulated in CNF1-treated BMDMs compared to those in dialysis buffer-treated BMDMs (*P < *0.01 and fold change > 2) (Fig. 2A). Kyoto Encyclopedia of Genes and Genomes (KEGG) pathway analysis revealed that the upregulated genes were mainly enriched in NF-κB, MAPK, and JAK-STAT signaling pathways, which are involved in M1 macrophage polarization regulation (Fig. 2B). To determine the exact pathway that contributes to CNF1-mediated M1 macrophage polarization, CNF1-induced BMDMs were exposed to NF-κB inhibitor (Bay 11-7085), JAK1/2 inhibitor (AZD1480), ERK inhibitor (SCH772984), p38 inhibitor (SB 239063), or JNK inhibitor (JNK-IN-8), respectively. We found that most of the M1 signature proinflammatory cytokine mRNA levels induced by CNF1 were partially decreased by NF-κB or JAK1/2 inhibitor (Fig. 2C and D), whereas the mRNA levels of these cytokines were not obviously changed when CNF1-induced BMDMs were challenged with the three kinds of MAPK inhibitors (see Fig. S1F to H). In addition, the phosphorylations of IκBα (pIκBα) and JAK1 (pJAK1) were increased in CNF1-treated BMDMs or human monocyte cell line THP1-derived macrophages (Fig. 2E to G). We also examined the phosphorylated protein of STAT1 (pSTAT1), STAT3 (pSTAT3), and STAT5 (pSTAT5), which are involved in JAK-STAT-related M1 macrophage polarization, and found that pSTAT1, but not pSTAT3 or pSTAT5, was increased in CNF1-treated BMDMs or THP1-derived macrophages (Fig. 2H and I; see also Fig. S1I and J). CNF1-induced mRNA levels of M1 cytokines were also partially decreased by pSTAT1 inhibitor (Fludarabine) (Fig. 2J). Moreover, flow cytometry analysis revealed that pretreatment with NF-κB, JAK1/2, or pSTAT1 inhibitor significantly inhibited CNF1-induced M1 macrophage polarization, whereas no effect was observed for M2 macrophage polarization (Fig. 2K and L; see also Fig. S1K to M). In addition, we found that pretreatment with NF-κB attenuated CNF1-mediated pSTAT1 increasement (see Fig. S1N) and that pSTAT1 inhibitor affected CNF1-mediated NF-κB activation (see Fig. S1O); however, JAK1/2 inhibitor only affected CNF1-mediated pSTAT1 increasement but not NF-κB activation (see Fig. S1P and Q). These results suggest that NF-κB and STAT1 signaling also activated each other to enhance the effect of CNF1. Taken together, these results indicate that CNF1 enhances M1 macrophage polarization through NF-κB and JAK-STAT1 signaling pathways. To eliminate the possibility that LPS contained in purified CNF1 impacted the phenotypes driven by CNF1, the endotoxin level of the recombinant CNF1 additionally purified by size exclusion chromatography was determined using the ToxinSensor™ Chromogenic LAL Endotoxin assay kit. CNF1 contained less than 0.0003 ng/mL of endotoxin (see Fig. S2A to C), which failed to induce cytokines production in macrophages, as previously reported (30, 31). Moreover, we examined whether YadC, a UPEC fimbria protein purified similarly as CNF1, affects macrophage polarization in BMDMs. The results showed that the percentage of M1 and M2 macrophages were similar in BMDMs treated with YadC and buffer (see Fig. S2D). Accordingly, mRNA levels of M1- or M2-characterized markers are not different in the YadC and buffer groups (see Fig. S2E). In addition, YadC did not activate NF-κB and JAK-STAT1 signaling pathways (see Fig. S2F). Together, the effects of CNF1 on M1 macrophage polarization are unrelated to lipopolysaccharide (LPS).

10.1128/mbio.01147-22.2 FIG S2 Inhibition of Rho GTPase Rac1 impacts macrophage polarization induced by CNF1. (A) Size exclusion chromatogram analysis of purified CNF1 protein. (B) Chromatographic fractions resolved on SDS-PAGE and visualized by staining using Coomassie brilliant blue. (C) Concentrations of endotoxin in purified CNF1 protein. (D to F) BMDMs were treated with YadC (3 nM) and dialysis buffer for 6 h (*n* = 3). (D) Flow cytometry analysis showing the percentages of M1 and M2 macrophages. (E) mRNA levels of M1-characterized markers *Tnf-α*, *Il-6*, *Il-12*, *iNos*, and *Il-1β* (left) and mRNA levels of M2-characterized markers *Tgf-β*, *Ym1*, *Il-10*, *Mrc1*, and *Pparγ* (right). (F) Western blotting of pJAK1/JAK1, pSTAT1 (Tyr701)/STAT1, and pIκBα (Ser32)/IκBα protein levels in YadC-treated BMDMs. (G to I) Effect of Rac1 inhibitor (EHT 1864), Cdc42 inhibitor (ML141), or RhoA inhibitor (CCG-1423) on mRNA levels of *Tnf-α*, *Il-6*, *Il-12*, *iNos*, and *Il-1β* in the indicated BMDMs (*n* = 3). (J to L) Effect of Rac1 inhibitor (EHT 1864), Cdc42 inhibitor (ML141), or RhoA inhibitor (CCG-1423) on the percentages of M1 or M2 macrophages in the indicated BMDMs (top) (*n* = 3). Representative flow cytometry dot plots (bottom) are shown. (M and N) Western blot showing the effect of Rac1 inhibitor (EHT 1864) on pIκBα (Ser32)/IκBα (M) and pSTAT1 (Tyr701)/STAT1 (N) in the indicated BMDMs. The data represent the means ± the SEM. A Student *t* test (D and E) or one-way ANOVA (G to L) was performed (*, *P* < 0.05; **, *P* < 0.01; ***, *P* < 0.001; ****, *P* < 0.0001). Download FIG S2, TIF file, 2.8 MB. Copyright © 2022 Sun et al. 2022 Sun et al. https://creativecommons.org/licenses/by/4.0/ This content is distributed under the terms of the Creative Commons Attribution 4.0 International license.

### CNF1 regulates the NF-κB and JAK-STAT1 signaling pathways partially through Rac1.

CNF1 is known to activate Rho GTPases (11). To examine whether CNF1 induces NF-κB and JAK-STAT1 signaling pathways and M1 macrophage polarization through activating Rho GTPases, Rac1, Cdc42, or RhoA inhibitor (EHT 1864, ML141, or CCG-1423) was used to treat BMDMs. Most of the CNF1-induced M1 gene expression was diminished by pretreatment with Rac1 inhibitor; whereas these cytokine mRNA levels were not obviously restored by Cdc42 or RhoA inhibitor (see Fig. S2G to I). In addition, CNF1-mediated M1 macrophage polarization was partially restored by Rac1 inhibitor but not by Cdc42 or RhoA inhibitor (see Fig. S2J to L). Moreover, pretreatment with Rac1 inhibitor also partially restored CNF1-activated NF-κB and JAK-STAT1 signaling pathways (see Fig. S2M and N). These results reveal that CNF1-activated Rac1 is partially involved in CNF1-mediated NF-κB and JAK-STAT1 signaling pathway activation and M1 macrophage polarization.

### CNF1 physically interacts with JAK1.

**FIG 3:**
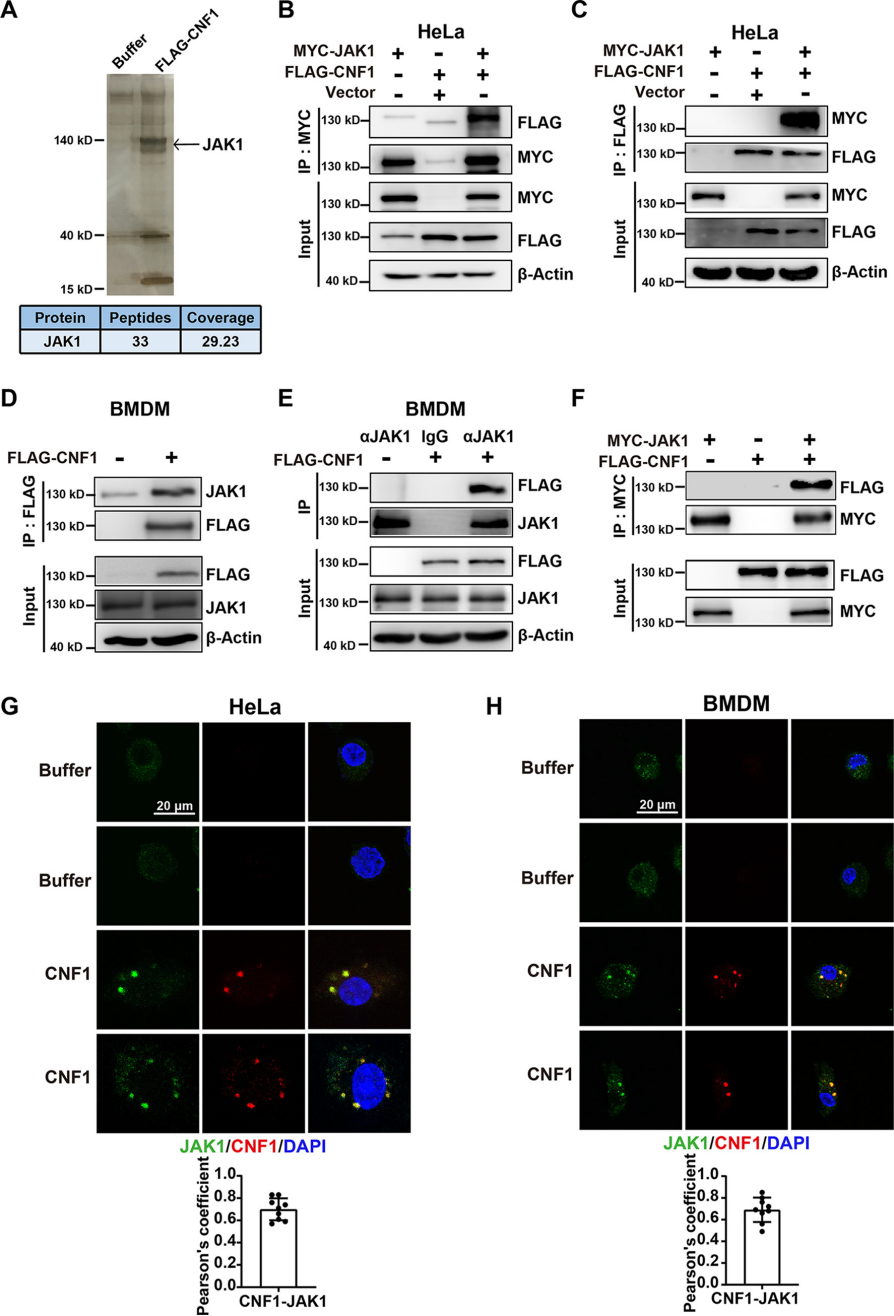
CNF1 physically interacts with JAK1. (A) Silver staining and mass spectrometry analysis of CNF1-associated proteins after immunoprecipitation in the indicated BMDMs. (B and C) Co-IP analysis of the interaction between recombinant FLAG-tagged CNF1 and recombinant MYC-tagged JAK1 transfected in HeLa cells. (D and E) Co-IP analysis of the interaction between recombinant FLAG-tagged CNF1 and endogenous JAK1 in BMDMs. α, anti-. (F) Co-IP analysis of the interaction between recombinant FLAG-tagged CNF1 and recombinant MYC-tagged JAK1 protein *in vitro*. (G and H) Representative immunofluorescence images of recombinant FLAG-tagged CNF1 and endogenous JAK1 in HeLa cells and BMDMs (top). Pearson’s coefficients (*R* values) for CNF1-JAK1 colocalization (bottom) in HeLa cells and BMDMs were determined (*n* = 9 random areas per group from three independent experiments). Scale bar, 20 μm. Colors: FLAG-tagged CNF1, red; DAPI, blue; and endogenous JAK1, green.

Since Rac1 inhibition did not completely restore CNF1-induced M1 polarization and C866S mutant (eliminating CNF1’s effect on Rho GTPases activation [12]) still activated JAK-STAT1 signaling pathway and promoted M1 macrophage polarization (see Fig. S3A to F), we hypothesized that CNF1 could interact with other host proteins except Rho GTPases. Recombinant FLAG-tagged CNF1 or dialysis buffer was incubated with BMDMs, and immunoprecipitation (IP) with FLAG antibody was performed with BMDM extracts. Specific proteins in the CNF1 group were analyzed by using liquid chromatography-tandem mass spectrometry (LC-MS/MS), and the key protein in the JAK-STAT signaling pathways, JAK1, was identified (Fig. 3A). To further confirm the association between CNF1 and JAK1, coimmunoprecipitation (co-IP) assays using HeLa cells transfected with MYC-tagged JAK1 and incubated with recombinant FLAG-tagged CNF1 were carried out. JAK1 was immunoprecipitated with recombinant FLAG-tagged CNF1 and vice versa (Fig. 3B and C). An association between recombinant FLAG-tagged CNF1 and endogenous JAK1 in BMDMs was reciprocally verified by co-IP (Fig. 3D and E). In addition, direct interaction between CNF1 and JAK1 was demonstrated by performing *in vitro* pulldown experiments using purified recombinant FLAG-tagged CNF1 and MYC-tagged JAK1 (Fig. 3F). Moreover, colocalization of CNF1 and endogenous JAK1 in living HeLa cells and BMDMs was observed by using immunofluorescence assays (Fig. 3G to H). These results together indicated that CNF1 directly interacts with JAK1, which may affect JAK-STAT1 pathways and M1 macrophage polarization.

10.1128/mbio.01147-22.3 FIG S3 Membrane translocation domain of CNF1 interacts with JAK1. (A to E) BMDMs were treated with C866S (3 nM) and dialysis buffer for 6 h (*n* = 3). (A) Flow cytometry analysis showing the percentages of M1 and M2 macrophages. (B) mRNA levels of M1-characterized markers *Tnf-α*, *Il-6*, *Il-12*, *iNos*, and *Il-1β* (left) and mRNA levels of M2-characterized markers *Tgf-β*, *Ym1*, *Il-10*, *Mrc1*, and *Pparγ* (right). (C to E) Effect of NF-κB inhibitor (Bay 11-7085) (C), JAK1/2 inhibitor (AZD1480) (D), and STAT1 inhibitor (Fludarabine) (E) on mRNA levels of *Tnf-α*, *Il-6*, *Il-12*, *iNos*, and *Il-1β* in the indicated BMDMs. (F) Western blot of pJAK1/JAK1, pSTAT1 (Tyr701)/STAT1, and pIκBα (Ser32)/IκBα protein levels in C866S-treated BMDMs or THP-1 cells. (G, I, and J) BMDMs were treated with FLAG-tagged CNF1 (3 nM), C866S (3 nM), N2 (3 nM), and dialysis buffer for 6 h. (G) Representative immunofluorescence images show the interaction of FLAG-tagged N2 or dialysis buffer and endogenous JAK1 in BMDMs (left). Pearson’s coefficients (*R* values) for N2-JAK1 colocalization (right) in BMDMs are also shown (*n* = 9 random areas per group from three independent experiments). Scale bar, 15 μm. Colors: FLAG-tagged N2, red; DAPI, blue; and endogenous JAK1, green. (H) Co-IP analysis of recombinant FLAG-tagged CNF1 and recombinant MYC-tagged PTPN6/GRB2. (I) Representative flow cytometry dot plots of the indicated BMDMs. (J) Flow cytometry analysis showing the percentages of M2 macrophages in BMDMs (*n* = 3). The data represent the means ± the SEM. A Student *t* test (A, B, and G) or one-way ANOVA (C to E and J) was performed (*, *P* < 0.05; **, *P* < 0.01; ***, *P* < 0.001; ****, *P* < 0.0001). Download FIG S3, TIF file, 3.0 MB. Copyright © 2022 Sun et al. 2022 Sun et al. https://creativecommons.org/licenses/by/4.0/ This content is distributed under the terms of the Creative Commons Attribution 4.0 International license.

### Molecular interactions between CNF1 and JAK1.

**FIG 4:**
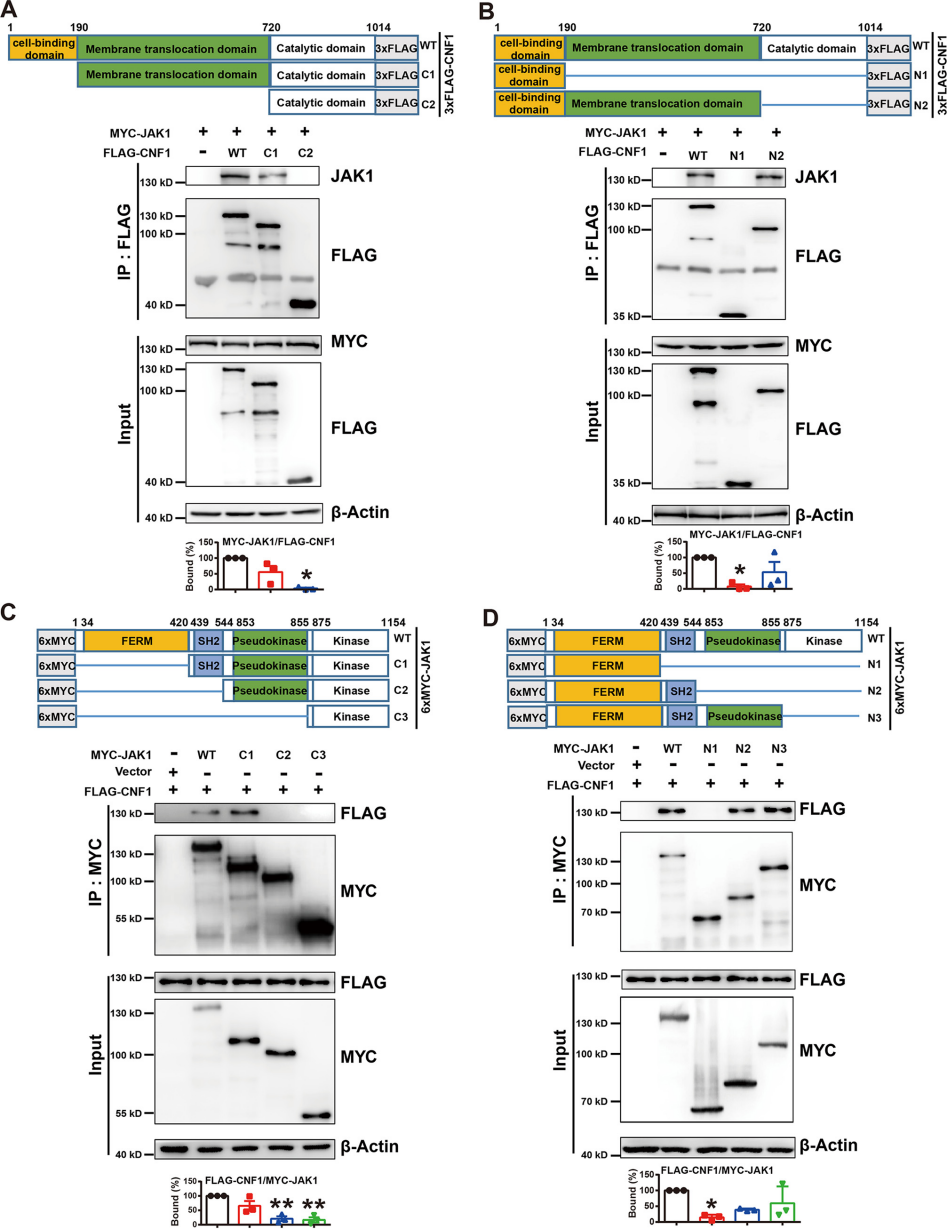
Molecular interactions between CNF1 and JAK1. (A and B) Schematic diagrams of FLAG-tagged CNF1 truncation mutants used in this study (top). 3×FLAG, 3×FLAG tag. Co-IP analysis of recombinant MYC-tagged JAK1 and truncated FLAG-tagged CNF1 proteins was performed (bottom). The density of Myc-tagged JAK1 was normalized to that of truncated FLAG-tagged CNF1 in the IP group. The percentage of bound MYC-tagged JAK1 to FLAG-tagged full-length CNF1 was set to 100% (*n* = 3). (C and D) Schematic diagram of MYC-tagged JAK1 truncation mutants used in this study (top). 6×MYC, 6×MYC tag. Co-IP analysis of recombinant FLAG-tagged CNF1 and truncated MYC-tagged JAK1 proteins was performed (bottom). The density of FLAG-tagged CNF1 was normalized to that of truncated Myc-tagged JAK1 in the IP group. The percentage of bound FLAG-tagged CNF1 to MYC-tagged full-length JAK1 was set to 100% (*n* = 3). The data represent the means ± the SEM. One-way ANOVA (A to D) was performed (*, *P* < 0.05; **, *P* < 0.01).

Three functional domains of CNF1, including cell binding, membrane translocation, and catalytic domains and four functional domains of JAK1, including kinases, FERM, SH2-like, and pseudokinase domains, have been reported (Fig. 4). In order to map the interface of CNF1-JAK1 interaction in detail, a series of truncation mutants of MYC-JAK1 and FLAG-CNF1 were genetically engineered, and interactions of purified truncated proteins were examined by using *in vitro* co-IP assays. The assays indicated that FLAG-CNF1 truncation mutants, including mutants 190-1014 (C1) and 1-720 (N2) but not mutants 720-1014 (C2) and 1-190 (N1), were able to bind to MYC-JAK1 (Fig. 4A and B). These results suggest that CNF1 interacted with JAK1 through the membrane translocation domain of CNF1 (mutant 190-720). Immunofluorescence assays also revealed strong colocalization of endogenous JAK1 and N2 in BMDMs (see Fig. S3G). In addition, MYC-JAK1 truncation mutants, including mutants 420-1154, 1-544, and 1-855 but not mutants 544-1154, 855-1154, and 1-420, were able to bind to FLAG-CNF1 (Fig. 4C and D), suggesting that JAK1 interacted with CNF1 through the SH2 domain of JAK1 (mutant 420-544). We further tested whether the interaction of CNF1 and JAK1’s SH2 domain is specific. A co-IP experiment between CNF1 and PTPN6/GRB2 containing the SH2 domain was performed. There was no interaction between CNF1 and PTPN6/GRB2, indicating a specific interaction of CNF1 and JAK1’s SH2 domain (see Fig. S3H). Collectively, these results suggest that the membrane translocation domain of CNF1 (mutant 190-720) and the SH2 domain of JAK1 (mutant 420-544) is necessary for CNF1-JAK1 interaction, which may be responsible for JAK-STAT1 activation and M1 macrophage polarization.

### CNF1 activated JAK-STAT1 signaling pathway through a CNF1-JAK1-JAK2 complex.

**FIG 5:**
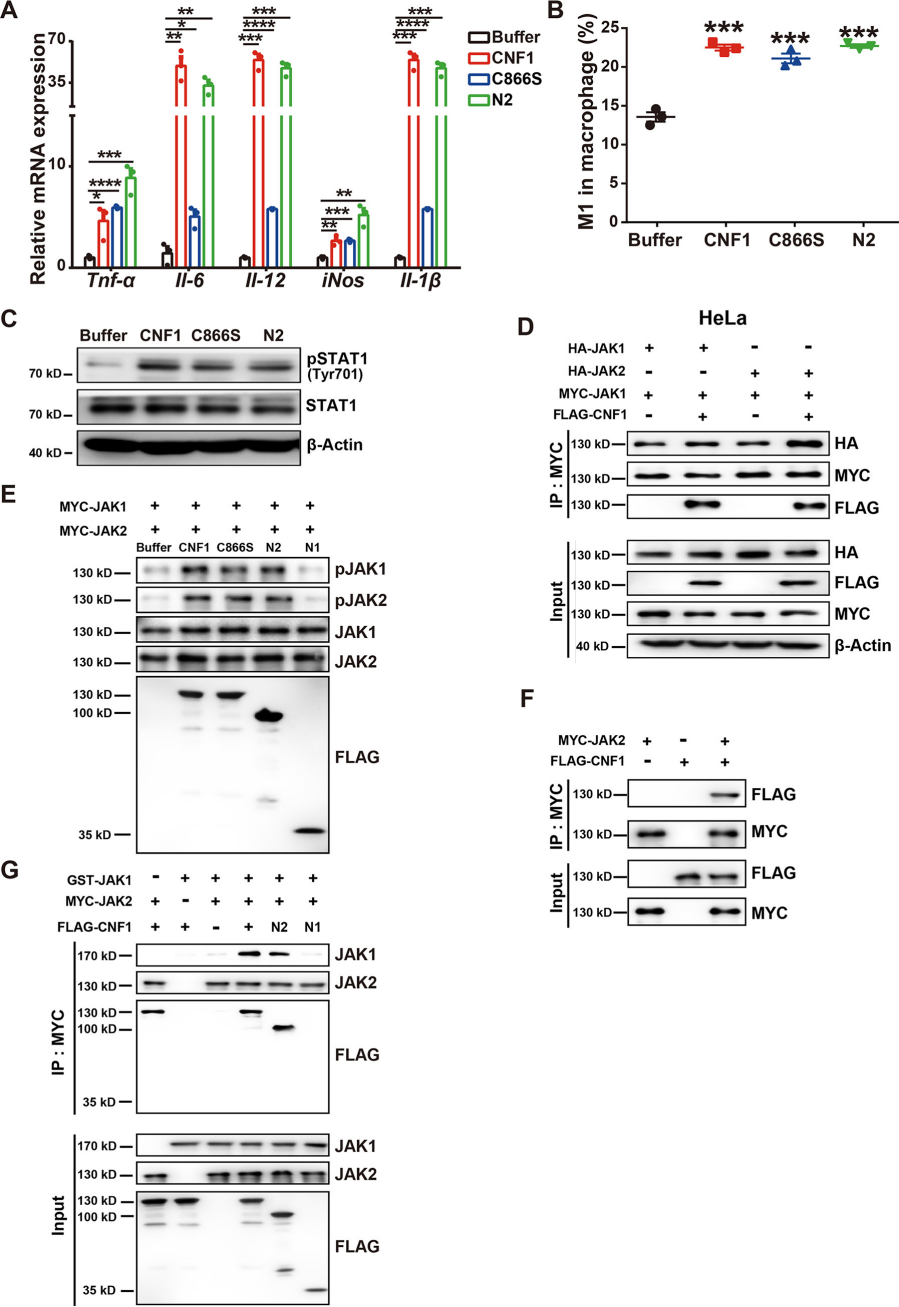
Membrane translocation domain of CNF1 promotes M1 macrophage polarization and directly activates JAK1 and JAK2. (A to C) BMDMs were treated with CNF1 (3 nM), C866S (3 nM), N2 (3 nM), and dialysis buffer for 6 h (*n* = 3). (A) mRNA levels of *Tnf-α*, *Il-6*, *Il-12*, *iNos*, and *Il-1β* in the indicated BMDMs. (B) Flow cytometry analysis results show the percentages of M1 macrophages in the indicated BMDMs. (C) Western blot of pSTAT1 (Tyr701)/STAT1 protein levels in the indicated BMDMs. (D) Co-IP analysis of the interaction between JAK1 and JAK1 or JAK2 in indicated HeLa. (E) *In vitro* kinase assays of JAK1 and JAK2 phosphorylation in the presence of FLAG-tagged CNF1 and various mutant CNF1s. (F) Co-IP analysis of the interaction between recombinant FLAG-tagged CNF1 and MYC-tagged JAK2 protein *in vitro*. (G) Co-IP analysis of the interaction between recombinant GST-tagged JAK1 and MYC-tagged JAK2 in the presence of FLAG-tagged CNF1 and various mutant CNF1s *in vitro*. The data represent the means ± the SEM. One-way ANOVA (A to C) was performed (*, *P* < 0.05; **, *P* < 0.01; ***, *P* < 0.001; ****, *P* < 0.0001).

Since CNF1 truncation mutant 190-720 does not include the functional domain (mutant 720-1014) involved in Rho GTPases activation previously reported (32), we hypothesized that CNF1 bound to JAK1 and induces its activation through a Rho GTPase-independent mechanism. The mRNA levels of *Tnf-α*, *Il-6*, *Il-12*, *iNos*, and *Il-1β* were significantly increased in BMDMs treated with CNF1, N2, or C866S (an inactive mutant in catalytic domain of CNF1) (Fig. 5A). Moreover, the percentages of M1 macrophages were significantly increased in BMDMs treated with CNF1, N2, or C866S (Fig. 5B; see also Fig. S3I in the supplemental material) but not for M2 macrophages (see Fig. S3J). In addition, pSTAT1 was obviously accumulated on BMDMs stimulated with CNF1, N2, or C866S (Fig. 5C; see also Fig. S4A). The expression levels of total STAT1 were confirmed to be comparable (Fig. 5C; see also Fig. S4B). Canonical M1 macrophage polarization is activated by IFN-γ and JAK1/2-STAT1 signaling pathways. Kinases JAK1 and JAK2 brings close proximity with one another, allowing them to transphosphorylate each other by IFN-γ-induced receptor oligomerization (33). Using co-IP assays in HeLa cells transfected with MYC-tagged JAK1 and hemagglutinin (HA)-tagged JAK1 or HA-tagged JAK2, we observed that the binding of JAK1 and JAK2, compared to that of JAK1 and JAK1, was obviously increased in cells treated with CNF1 (Fig. 5D). To demonstrate whether JAK1 and JAK2 transphosphorylate each other directly mediated by CNF1, *in vitro* kinase assays were performed in the presence of ATP, purified MYC-tagged JAK1, and purified MYC-tagged JAK2, in addition with CNF1, N1, N2, or C866S. Phosphorylation of both JAK1 and JAK2 was observed in the CNF1, N2, or C866S group compared to the N1 and control group (Fig. 5E). Moreover, CNF1 still induced STAT1 phosphorylation in the presence of IFN-γ neutralizing antibodies, indicating that CNF1-mediated JAK-STAT1 activation is independent of IFN-γ-mediated receptor oligomerization (see Fig. S4C). We further sought to determine whether CNF1 also interacts with JAK2 and brings JAK1 and JAK2 together through forming a protein complex. Using *in vitro* co-IP assays with purified proteins, direct interaction between CNF1 and JAK2 was observed (Fig. 5F). Moreover, a direct association of glutathione *S*-transferase (GST)-tagged JAK1 and MYC-tagged JAK2 was observed in the presence of CNF1 and N2 by using *in vitro* co-IP assays, indicating the formation of a CNF1-JAK1-JAK2 protein complex (Fig. 5G). Collectively, these results suggest that CNF1 (independent of its Rho GTPase activation) directly interacts with JAK1 and JAK2 to form a protein complex, which brings JAK1 and JAK2 into close proximity to induce their phosphorylation, followed by STAT1 activation and M1 macrophage polarization.

10.1128/mbio.01147-22.4 FIG S4 CNF1 undergoes LLPS relying on the IDRs domain to drive the formation of CNF1-JAK1-JAK2 complex. (A and B) BMDMs were treated with FLAG-tagged CNF1 (3 nM), C866S (3 nM), N2 (3 nM), and dialysis buffer for 6 h. (A) Representative immunofluorescence images of pSTAT1 (Tyr701) in the indicated BMDMs (top). Quantification of the fluorescence density of pSTAT1 (Tyr701) in indicated BMDMs was also performed (bottom). Scale bar, 40 μm. Colors: pSTAT1 (Tyr701), red; and DAPI, blue. (B) Representative immunofluorescence images of total STAT1 in the indicated BMDMs (top). Quantification of the fluorescence density of total STAT1 in indicated BMDMs was also performed(bottom). Scale bar, 40 μm. Colors: STAT1, green; and DAPI, blue. (C) Western blot showing the pSTAT1 (Tyr701)/STAT1 protein levels in THP-1 cells treated with anti-IFN-γ (50 μg) or anti-IgG (50 μg) and infected with FLAG-tagged CNF1 (3 nM) and dialysis buffer for 6 h. (D) Co-IP analysis of the interaction between recombinant GST-tagged CNF1^190-720^ or CNF1^190-720, IDRs^ and recombinant MYC-tagged JAK1/2. The data represent the means ± the SEM. One-way ANOVA (A and B) was performed (*, *P* < 0.05; **, *P* < 0.01). Download FIG S4, TIF file, 2.8 MB. Copyright © 2022 Sun et al. 2022 Sun et al. https://creativecommons.org/licenses/by/4.0/ This content is distributed under the terms of the Creative Commons Attribution 4.0 International license.

### CNF1 exhibits LLPS to form CNF1-JAK1-JAK2 complex.

**FIG 6:**
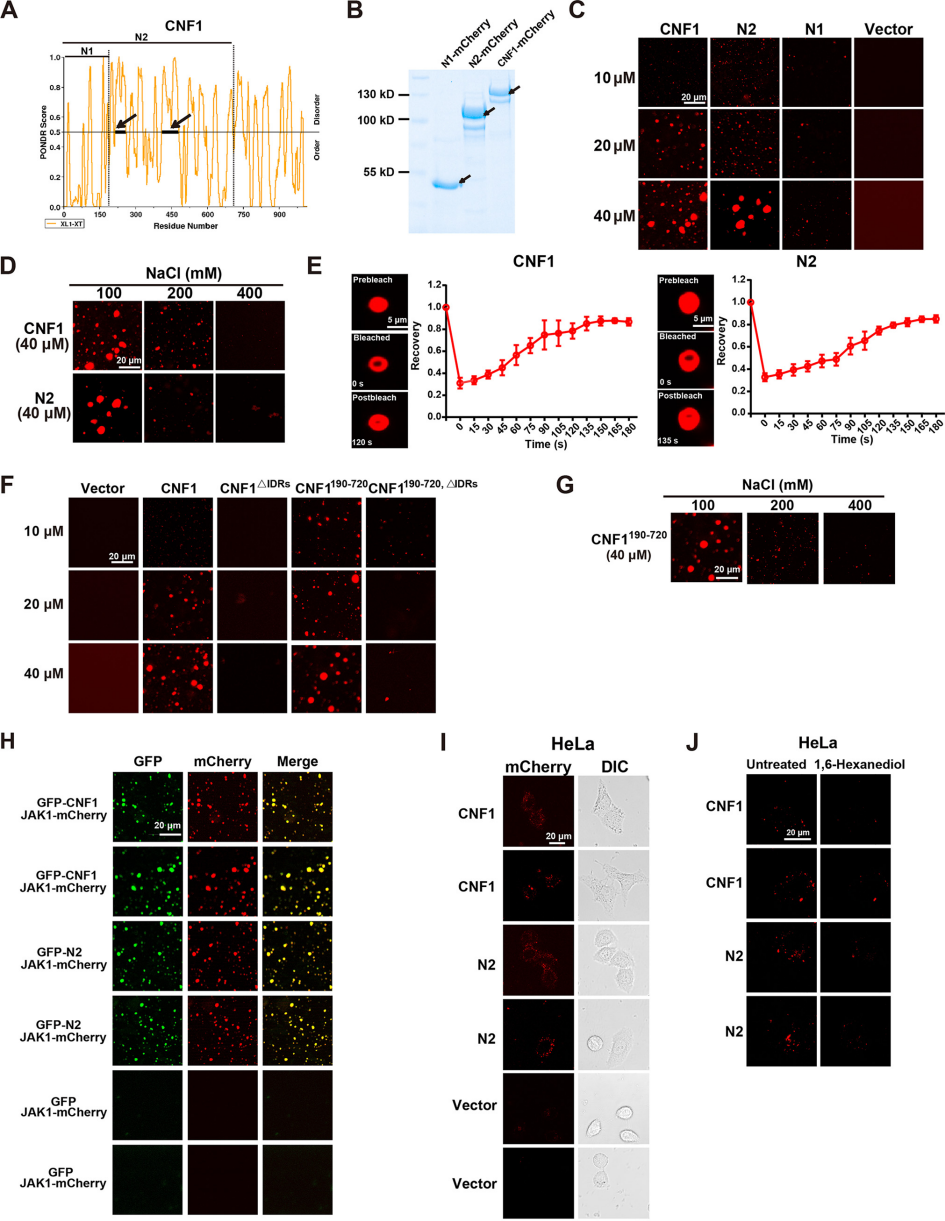
CNF1 and its membrane translocation domain enhances JAK1 and JAK2 liquid-liquid phase separation in BMDMs. (A) Prediction of intrinsic disorder of CNF1 protein using a XL1-XT algorithm. For PONDR prediction, a score above 0.5 indicates a high degree of disorder. Heavy bars indicate regions predicted to be intrinsically disordered. (B) mCherry-fusion CNF1, mCherry-fusion N2, and mCherry-fusion N1 proteins resolved on SDS-PAGE and detected by staining with Coomassie brilliant blue. (C) Representative immunofluorescence images of droplet formation at the indicated concentrations of proteins. mCherry-fusion CNF1, mCherry-fusion N2, mCherry-fusion N1, or mCherry was added to the droplet formation buffer with 100 mM NaCl and 10% PEG-8000. Scale bar, 20 μm. (D) Representative immunofluorescence images of mCherry-fusion CNF1 and mCherry-fusion N2 at 40 μM protein concentration droplet formation at different salt concentrations. Scale bar, 20 μm. (E) FRAP measurements of CNF1-mCherry and N2-mCherry droplets at the indicated times (left). Normalized FRAP intensity curves of CNF1-mCherry and N2-mCherry droplets (right). Scale bar, 5 μm. (F) Representative immunofluorescence images of droplet formation at the indicated concentrations of proteins. mCherry-fusion CNF1, mCherry-fusion CNF1^IDRs^, mCherry-fusion CNF1^190-720^, mCherry-fusion CNF1^190-720, IDRs^, or mCherry was added to the droplet formation buffer with 100 mM NaCl and 10% PEG-8000. Scale bar, 20 μm. (G) Representative immunofluorescence images of mCherry-fusion CNF1^190-720^ at a 40 μM protein concentration droplet formation at different salt concentrations. Scale bar, 20 μm. (H) Representative immunofluorescence images of puncta formation for GFP-fusion CNF1, GFP-fusion N2, or GFP with mCherry-fusion JAK1. Scale bar, 20 μm. (I and J) HeLa cells were treated with mCherry-fusion CNF1, mCherry-fusion N2, or mCherry for 6 h. (I) Representative immunofluorescence images of mCherry-fusion CNF1, mCherry-fusion N2, and mCherry puncta in HeLa cells. Scale bar, 20 μm. (J) Representative immunofluorescence images of mCherry-fusion CNF1 and mCherry-fusion N2 puncta in HeLa cells before and after treatment with 3% 1,6-hexanediol for 1 min. Scale bar, 20 μm (*n* = 4/group) (E). The data represent the means ± the SEM.

**FIG 7:**
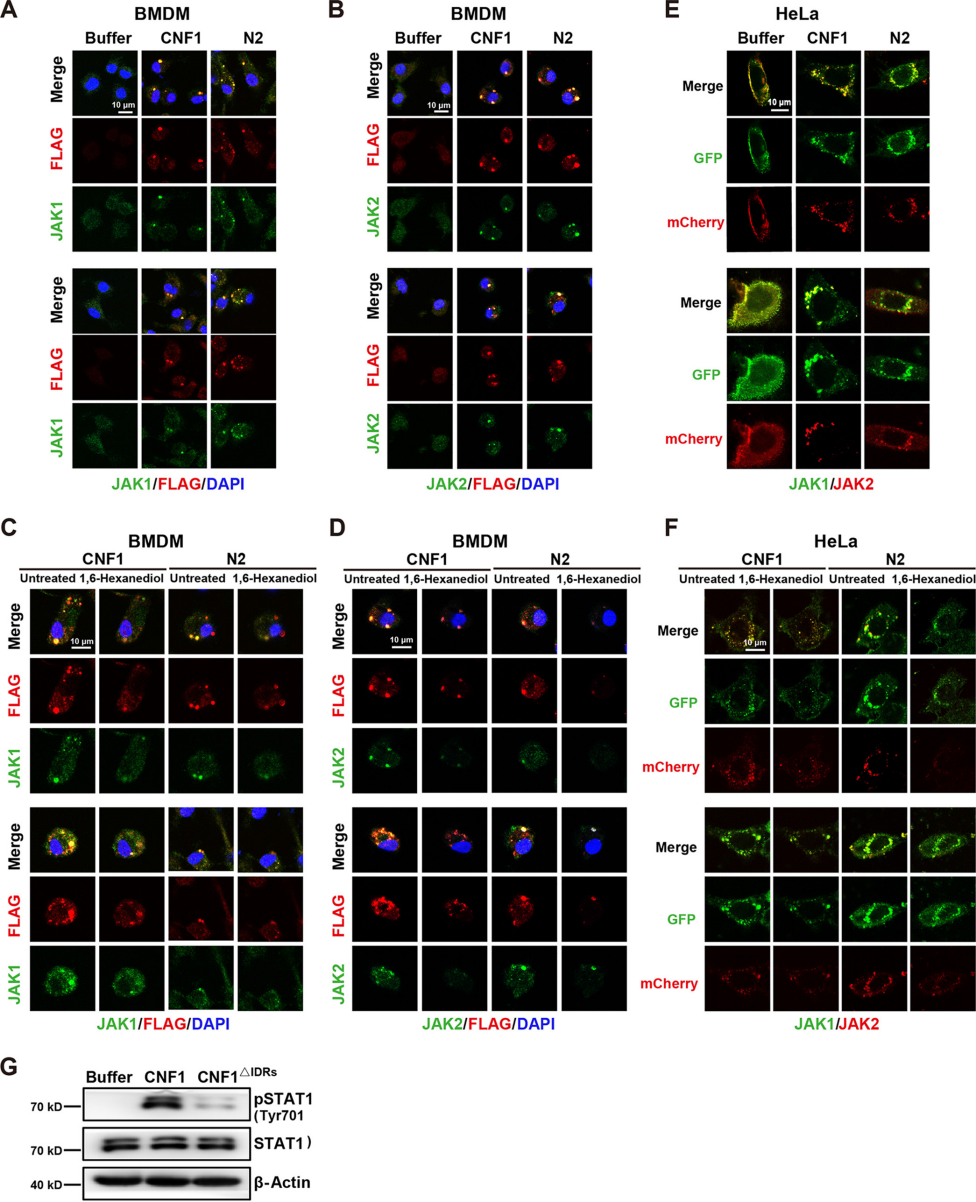
CNF1 undergoes LLPS to drive the formation of CNF1-JAK1-JAK2 complex in living cells. (A and B) BMDMs were treated with FLAG-tagged CNF1, N2, or dialysis buffer for 6 h. Representative immunofluorescence images of FLAG-tagged CNF1/N2 (3 nM) or dialysis buffer and endogenous JAK1 (A) or JAK2 (B) are shown. (C and D) Representative immunofluorescence images of FLAG-tagged CNF1 or N2 and endogenous JAK1 (C) or JAK2 (D) puncta in BMDMs before and after treatment with 3% 1,6-hexanediol for 10 min. Scale bar, 10 μm. Colors: FLAG-tagged CNF1 or N2, red; DAPI, blue; and endogenous JAK1 or JAK2, green. (E) Live cell immunofluorescence imaging for phase separation formation of HeLa cells coexpressing JAK1-GFP and JAK2-mCherry in CNF1, N2, or dialysis buffer-treated conditions. (F) Live cell immunofluorescence imaging of HeLa cells overexpressing JAK1-GFP and JAK2-mCherry in CNF1- or N2-treated conditions before and after treatment with 3% 1,6-hexanediol for 1 min. Scale bar, 10 μm. Colors: JAK2-mCherry, red; and JAK1-GFP, green. (G) Western blot of pSTAT1 (Tyr701)/STAT1 protein levels in the indicated BMDMs.

CNF1 was shown as discrete puncta rather than in a diffused status in host cells when used to treat HeLa cells or BMDMs (Fig. 3G and H). We wondered whether CNF1 undergoes LLPS. Interestingly, CNF1 contained two putative intrinsically disordered regions (IDRs) located in the membrane translocation domain (Fig. 6A). To test the ability of CNF1 and N2 phase separation, we purified CNF1-mCherry, N2-mCherry, and N1-mCherry proteins to measure droplet formation (Fig. 6B). Both CNF1-mCherry and N2-mCherry formed numerous micrometer-sized, spherical droplets in a concentration-dependent manner *in vitro*, whereas no obvious droplets were observed for N1-mCherry at the same concentration (Fig. 6C). Moreover, CNF1-mCherry and N2-mCherry droplets were abolished by increasing NaCl concentrations (Fig. 6D), and liquid-like recovery kinetics of CNF1-mCherry and N2-mCherry droplets were examined using FRAP (fluorescence recovery after photobleaching) analysis (Fig. 6E). These results indicate that CNF1-mCherry and N2-mCherry droplets are dynamic and reversible. To investigate whether these CNF1 IDRs promote CNF1 phase separation *in vitro*, we purified the IDR deletion mutants of CNF1 (CNF1^IDRs^ and CNF1^190-720, IDRs^) and a truncation mutant containing the IDRs (CNF1^190-720^). Droplet formation assays revealed that CNF1^IDRs^ and CNF1^190-720, IDRs^ failed to form droplets and that CNF1^190-720^ formed numerous micrometer-sized, spherical droplets in a concentration-dependent manner (Fig. 6F). Moreover, these micrometer-sized droplets in CNF1^190-720^ were eliminated by increasing NaCl concentrations (Fig. 6G). These results indicate that the IDR domain within CNF1 possesses the ability to drive CNF1 LLPS *in vitro*. We also examined the potential impact of the IDR domain upon interaction with JAK1/2. An interaction between CNF1^190-720, IDRs^ and JAK1/2 was observed (see Fig. S4D), suggesting that the IDR domain of CNF1 is not necessary for interaction with JAK1/2. We further evaluated whether CNF1 and N2 (containing the IDR domain) impact potential JAK1 phase separation *in vitro*. We purified the recombinant mCherry-JAK1 protein. Droplet formation assays revealed that JAK1 failed to form the condensed puncta in the absence of CNF1, whereas double-positive spherical droplets were observed in JAK1, together with CNF1 or N2 (Fig. 6H). This observation implies that CNF1 phase-separated condensates incorporate and concentrate JAK1 *in vitro*. Next, we sought to determine whether CNF1 proteins undergo phase separation in living cells. We used CNF1-mCherry or N2-mCherry to treat live HeLa cells, the puncta of CNF1 and N2 were observed in cells (Fig. 6I), and 1,6-hexanediol treatment obviously reduced the puncta (Fig. 6J). To examine whether JAK1/2 can spontaneously incorporate into CNF1 phase-separated condensates in living cells, FLAG-tagged CNF1, N2, or dialysis buffer was used to treat BMDMs. Endogenous JAK1 or JAK2 were colocalized within the CNF1 or N2 puncta (Fig. 7A and B), and the treatment of 1,6-hexanediol decreased these puncta (Fig. 7C and D). Moreover, increased puncta and colocalization of JAK1-GFP and JAK2-mCherry were observed in HeLa cells overexpressing JAK1-GFP and JAK2-mCherry treated with CNF1 and N2 compared to those treated with dialysis buffer (Fig. 7E), and 1,6-hexanediol treatment decreased these puncta and colocalization (Fig. 7F). These full-length CNF1 puncta begin to appear in the cytosol within a few minutes, and aggregates are widely distributed in the cytosol by 6 h (see Videos S1 and S2). Many CNF1 and N2 puncta were not localized in LAMP1^+^ lysosomes, EEA1^+^ endosomes, and LC3^+^ autophagosomes at 1 and 6 h (see Fig. S5A to C), implying the forming of a CNF1-JAK1-JAK2 membraneless complex in living cells. We further examined whether the complex affects downstream STAT1 phosphorylation in living cells. The result showed that CNF1 promoted the phosphorylation of STAT1, which was eliminated by deletion of the IDR domain (Fig. 7G), indicating that the IDR domain is essential for the formation of the CNF1-JAK1-JAK2 complex and subsequent STAT1 activation. Taken together, these results suggest that CNF1 undergoes LLPS, relying on the IDR domain to drive the formation of the CNF1-JAK1-JAK2 complex, thus promoting STAT1 activation.

10.1128/mbio.01147-22.5 FIG S5 CNF1 promotes the phosphorylation of STAT1 through the IDR domain. (A and B) HeLa cells were treated with FLAG-tagged CNF1, N2, or dialysis buffer for 1 and 6 h. Representative immunofluorescence images of FLAG-tagged CNF1/N2 (15 nM) or dialysis buffer and EEA1 (A) or LAMP1 (B) are shown. Scale bar, 10 μm. Colors: FLAG-tagged CNF1/N2, red; EEA1/LAMP1, green; and DAPI, blue. (C) At 48 h posttransfection with plasmids encoding LC3-EGFP, the HeLa cells were treated with FLAG-tagged CNF1, N2, or dialysis buffer for 1 and 6 h. Representative immunofluorescence images of FLAG-tagged CNF1/N2 (15 nM) or dialysis buffer and LC3 are shown. Scale bar, 10 μm. Colors: FLAG-tagged CNF1/N2, red; LC3, green; and DAPI, blue. Download FIG S5, TIF file, 2.5 MB. Copyright © 2022 Sun et al. 2022 Sun et al. https://creativecommons.org/licenses/by/4.0/ This content is distributed under the terms of the Creative Commons Attribution 4.0 International license.

10.1128/mbio.01147-22.8 VIDEO S1 Real-time visualization of CNF1 getting into the cytosol of HeLa cells within a few minutes. Download Movie S1, AVI file, 3.0 MB. Copyright © 2022 Sun et al. 2022 Sun et al. https://creativecommons.org/licenses/by/4.0/ This content is distributed under the terms of the Creative Commons Attribution 4.0 International license.

10.1128/mbio.01147-22.9 VIDEO S2 Real-time visualization of CNF1 getting into the cytosol of HeLa cells and forming condensed puncta at 6 h. Download Movie S2, AVI file, 4.4 MB. Copyright © 2022 Sun et al. 2022 Sun et al. https://creativecommons.org/licenses/by/4.0/ This content is distributed under the terms of the Creative Commons Attribution 4.0 International license.

### Macrophage elimination or NF-κB/JAK-STAT1 pathway inhibition protects against CNF1-mediated acute kidney injury.

**FIG 8:**
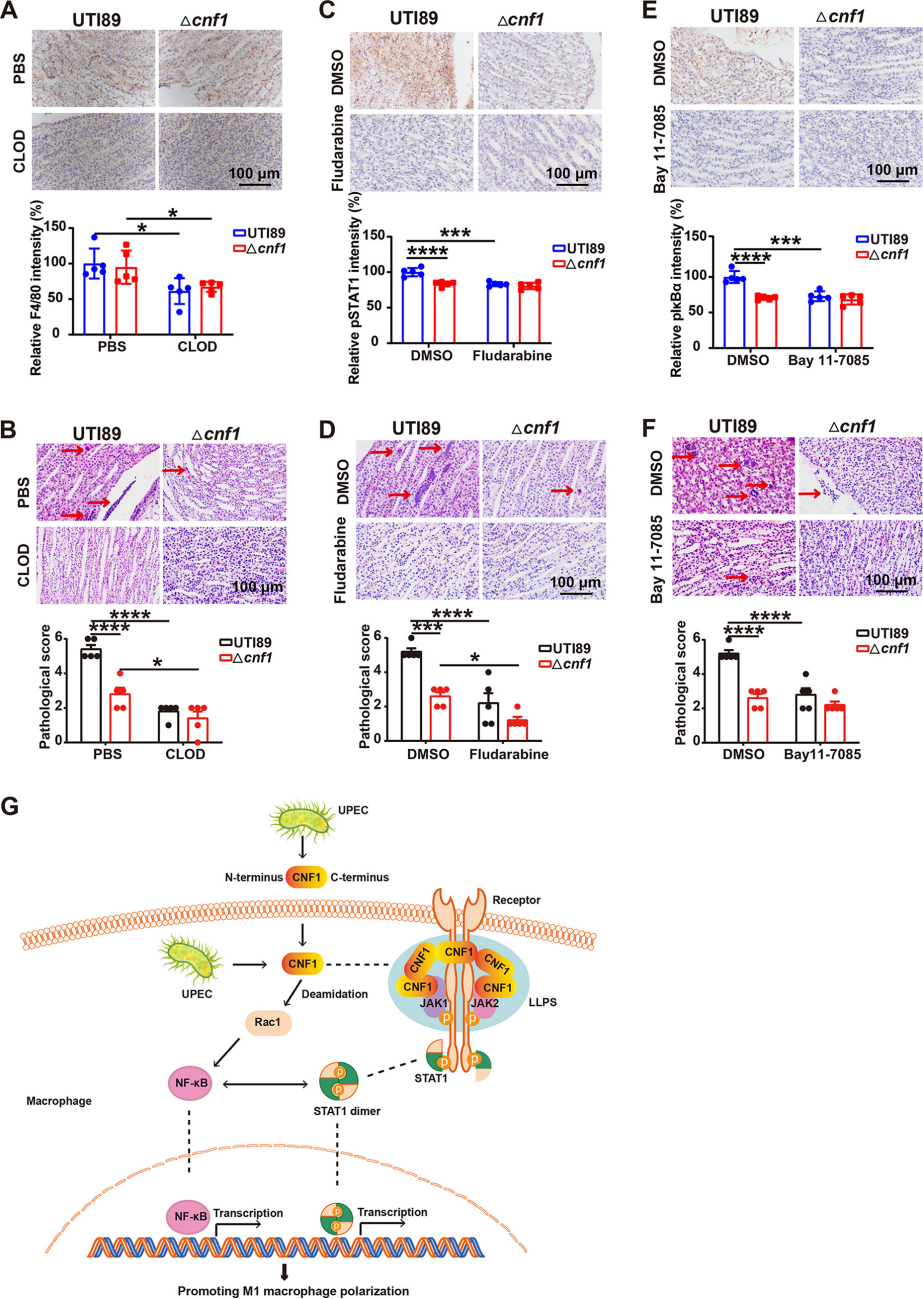
Macrophage elimination or inhibition of NF-κB or JAK-STAT1 pathway attenuates kidney injury induced by CNF1. (A) Immunohistochemistry staining of F4/80 in kidney tissues of female C57BL/6J mice treated with clodronate (CLOD) liposomes or PBS liposomes and infected with the UTI89 or Δ*cnf1* strain at 24 hpi (*n* = 5). (B) Hematoxylin and eosin (H&E) staining in representative images and histological scores of kidney tissues in female C57BL/6J mice treated with clodronate (Clod) liposomes or PBS liposomes and infected with the UTI89 or Δ*cnf1* strain at 24 hpi (*n* = 5). (C) Immunohistochemistry staining of pSTAT1 (Tyr701) in kidney tissues of female C57BL/6J mice treated with STAT1 inhibitor (Fludarabine) or control dimethyl sulfoxide (DMSO) and infected with the UTI89 or Δ*cnf1* strain at 24 hpi (*n* = 5). (D) H&E staining in representative images and histological scores of kidney tissues in female C57BL/6J mice treated with STAT1 inhibitor (Fludarabine) or control DMSO and the infected UTI89 or Δ*cnf1* strain at 24 hpi (*n* = 5). (E) Immunohistochemistry staining of pIκBα (Ser32) in kidney tissues of female C57BL/6J mice treated with NF-κB inhibitor (Bay 11-7085) or control DMSO and infected with the UTI89 or Δ*cnf1* strain at 24 hpi (*n* = 5). (F) H&E staining representative images and histological scores of kidney tissues in female C57BL/6J mice treated with NF-κB inhibitor (Bay 11-7085) or control DMSO and infected with the UTI89 or Δ*cnf1* strain at 24 hpi (*n* = 5). Scale bar, 100 μm. The arrows indicate papillary necrosis, tubular casts, and serious hemorrhage. (G) Graphical model illustrating the role of CNF1 in promoting M1 macrophage polarization. CNF1 produced by UPEC promoted M1 macrophage polarization through regulating NF-κB and JAK1/2-STAT1 signaling pathways. In addition to activating Rac1, CNF1 directly interacted with JAK1 and JAK2 to form a protein complex through LLPS, which induced JAK1/2 phosphorylation and the subsequent STAT1 activation. NF-κB and dimerized STAT1 migrated to the nucleus, where they bind to specific DNA-binding sites, regulating M1 macrophage polarization. The data represent the means ± the SEM. Two-way ANOVA was performed (*, *P* < 0.05; ***, *P* < 0.001; ****, *P* < 0.0001).

M1 macrophages exacerbate inflammation and tissue damage (34), and we previously reported that CNF1 enhanced acute kidney injury (13). To identify the role of increased M1 macrophages in CNF1-induced kidney injury, clodronate (Clod) liposomes (to eliminate macrophages) or PBS liposomes were injected intravenously into mice. The mice were then infected with UTI89 or Δ*cnf1* strains, respectively, at 24 h postinjection. The macrophage depletion was confirmed by immunohistochemical staining of F4/80 (Fig. 8A). The numbers and percentages of total macrophages and M1 macrophages in kidney tissues decreased markedly in the UTI89 or Δ*cnf1* group treated with Clod liposomes at 12 hpi, and no difference was noted for M1 macrophages between mice infected with UTI89 and Δ*cnf1* strains (see Fig. S6A and B). In addition, the pathological scores of kidneys were reduced in mice treated with Clod liposomes compared to mice treated with PBS liposomes when infected with the UTI89 or Δ*cnf1* strain, and no difference was observed between the UTI89 and Δ*cnf1* groups treated with Clod liposomes (Fig. 8B). Therefore, CNF1-induced M1 macrophages play a role in kidney injury. Since we demonstrated that CNF1 impacts macrophage polarization through NF-κB and JAK-STAT1 signaling pathways, we evaluated the effect of NF-κB or STAT1 inhibitor on CNF1-induced M1 macrophage polarization and kidney injury *in vivo*. Bay 11-7085 or Fludarabine was injected intraperitoneally into mice every day for 3 days, and the mice were infected with the UTI89 or *Δcnf1* strain, respectively. The effects of Bay 11-7085 and Fludarabine were validated by immunohistochemical staining of pSTAT1 (Tyr701) and pIκBα (Ser32) (Fig. 8C and E). Pretreatment with Bay 11-7085 or Fludarabine decreased the percentages and numbers of M1 macrophages in mice infected with UTI89 but had no effect on mice infected with the *Δcnf1* strain (see Fig. S6C to F). Moreover, no difference in M1 macrophages in kidneys was found between mice of the UTI89 and Δ*cnf1* groups injected with Bay 11-7085 or Fludarabine (see Fig. S6C to F). In addition, kidney injuries in mice infected with UTI89 were decreased after treatment with either Bay 11-7085 or Fludarabine, and no differences in kidney injuries were detected between mice of the UTI89 and Δ*cnf1* groups after Bay 11-7085 or Fludarabine injection (Fig. 8D to F). Collectively, these results indicate that CNF1-induced acute kidney injury depends on M1 macrophage polarization through NF-κB and JAK-STAT1 signaling pathways.

10.1128/mbio.01147-22.6 FIG S6 Inhibition of JAK-STAT1 and NF-κB pathway decreases M1 macrophages in kidneys induced by CNF1. (A and B) Female C57BL/6J mice were treated with clodronate (Clod) liposomes or PBS liposomes and infected with 10^8^ CFU of the UTI89 or Δ*cnf1* strain at 12 hpi (*n* = 5). (A) Representative flow cytometry dot plots of macrophages in kidneys (left). Flow cytometry analysis shows the percentages and numbers of macrophages in the kidneys (right). (B) Representative flow cytometry dot plots of M1 macrophages in kidneys (left). Flow cytometry analysis shows the percentages and numbers of M1 macrophages in kidneys (right). (C to F) Female C57BL/6J mice were treated with STAT1 inhibitor (Fludarabine), NF-κB inhibitor (Bay 11-7085), or DMSO and infected with 10^8^ CFU of the UTI89 or Δ*cnf1* strain at 12 hpi (*n* = 5). (C and E) Representative flow cytometry dot plots of macrophages in kidneys (left). Flow cytometry analysis shows the percentages and numbers of macrophages in kidneys (right). (D and F) Representative flow cytometry dot plots of M1 macrophages in kidneys (left). Flow cytometry analysis shows the percentages and numbers of M1 macrophages in kidneys (right). The data represent the means ± the SEM. Two-way ANOVA (A to F) was performed (*, *P* < 0.05; **, *P* < 0.01; ***, *P* < 0.001; ****, *P* < 0.0001). Download FIG S6, TIF file, 2.1 MB. Copyright © 2022 Sun et al. 2022 Sun et al. https://creativecommons.org/licenses/by/4.0/ This content is distributed under the terms of the Creative Commons Attribution 4.0 International license.

## DISCUSSION

In the present study, we elucidated an important role of CNF1 in macrophage reprogramming. We demonstrated that CNF1 not only partially regulated NF-κB and JAK-STAT1 signaling pathway through activating Rac1 but also directly interacted with JAK1 and JAK2 to form a protein complex through LLPS, consequentially promoting M1 macrophage polarization and subsequently inducing inflammatory response in kidney at an early stage of acute UTIs (Fig. 8G).

CNF1 is composed of three domains: the N-terminal cell-binding domain (amino acids 1 to 190), the membrane translocation domain (amino acids 190 to 720), and the C-terminal catalytic domain (amino acids 720 to 1014) (35). It is known that the catalytic domain of CNF1 modifies several Rho GTPases in the host cell cytosol (36). Surprisingly, we found that the CNF1-JAK1/2 interaction region (amino acids 190 to 720) lied in this membrane translocation domain. Nevertheless, one study demonstrates that full-length CNF1 can be detected in the cytosolic fraction of cells (37). In the present study, we found that full-length CNF1 and truncated CNF1 (N2, amino acids 1 to 720) were widely distributed in the cytosol, suggesting that CNF1 could be released from the endosomes to the cytosol without cleavage, but the mechanism still needs to be clarified. In addition, intracellular UPEC could directly secretes full-length CNF1 into host cell cytosol (38). Hence, it is likely that CNF1 interacts with JAK1/2 through its membrane translocation domain in the cytosol.

It has been demonstrated that the C-terminal catalytic domain (amino acids 720 to 1014) interferes with Rho GTPases (Rho, Rac, and Cdc42) (39). CNF1 deamidates a specific glutamine residue located in the *switch* 2 domain of G proteins (glutamine 63 in RhoA or glutamine 61 in Cdc42 and Rac1) (40), and this modification results in the constitutive activation of these proteins on their effectors, which are involved in several cellular processes, such as the modulation of cytoskeletal dynamics, gene transcription, cell adhesion, cell migration, cell polarity, and cell cycle progression (41). In the present study, we found that CNF1-activated Rac1 was partially involved in CNF1-mediated NF-κB and JAK-STAT1 signaling pathway activation and M1 macrophage polarization. Moreover, M1 macrophage polarization and the JAK-STAT1 signaling pathway were activated by C866S (without Rho GTPase activation), while activation of NF-κB signaling pathways by C866S was not observed. Considering that NF-κB and STAT1 signaling also activated each other to enhance the effect of CNF1, we speculated that the effect of CNF1 on Rac1 activation mainly induced NF-κB signaling pathway and also subsequently influenced the JAK-STAT1 signaling pathway, resulting in M1 macrophage polarization.

In the conventional scenario, cytokine-induced receptor aggregation leads to JAK1 and JAK2 proximity to phosphorylate JAK1/2 and associated receptors (42). The phosphorylation of tyrosine residues within the cytoplasmic tail of the receptor in turn provides docking sites for STAT1 through the Src homology 2 domain and leads to STAT1 phosphorylation, dimerization, and subsequent migration into the nucleus, regulating M1 macrophage polarization (43, 44). Interestingly, we found that CNF1 directly induced JAK1/2 phosphorylation without the presence of receptor. Moreover, we found that CNF1 exhibits LLPS phenomenon to induce CNF1-JAK1/2 complex. Several studies reveal that multivalent interactions driven by LLPS appear to be a general mechanism for signaling transduction (45–47). Therefore, we concluded that CNF1 interacted with JAK1/JAK2 and formed the CNF1-JAK1/2 complex through LLPS, which at least partly accounts for the increased JAK1/2 phosphorylation. JAK1/2 phosphorylation subsequently phosphorylates the cytoplasmic tail of associated receptors, which in turn recruits and activates STAT1. It is novel that CNF1 induces macrophage reprogramming independent of the conventional receptor aggregation.

Several studies show that the coordinated actions of membraneless condensates assembled via LLPS are involved in a wide variety of cellular processes, including regulating chromatin structure, gene expression, protein degradation, and signaling transduction (48–50). The protein-protein interaction by LLPS in the cytoplasm, nucleoplasm, and mitochondrial matrix have attracted much attention in recent years (51–53). Recent studies reveal that LLPS of viral proteins are implicated in a wide array of different steps and regulatory processes, including viral replication cycles and control of virus-host interactions (54). In addition, a recent study reports that the Xanthomonas campestris XopR exhibits LLPS to hijack *Arabidopsis* actin cytoskeleton (29). Nevertheless, LLPS of virulence factors from human-pathogenic bacteria and its functional consequences during pathogen-host interactions remain unclear. Here, we found that UPEC toxin CNF1 interacted with host JAK1/2 via LLPS to promote macrophage reprogramming.

## MATERIALS AND METHODS

### Bacterial strains.

Bacterial strains are listed in Table S1 in the supplemental material. UPEC strains were cultured at 37°C in Luria-Bertani (LB) medium containing 50 μg/mL kanamycin under static conditions for 12 h. UTI89 and a *cnf1* deletion strain derived from UTI89 (Δ*cnf1*) had been constructed as described previously (13).

10.1128/mbio.01147-22.7 TABLE S1 Materials used in this study. Download Table S1, PDF file, 0.3 MB. Copyright © 2022 Sun et al. 2022 Sun et al. https://creativecommons.org/licenses/by/4.0/ This content is distributed under the terms of the Creative Commons Attribution 4.0 International license.

### Mice.

All wild-type female C57BL/6J mice, aged 6 to 8 weeks, were purchased from Academy of Military Medical Science (Beijing, China). All mice were maintained under specific-pathogen-free conditions with a 25°C room temperature and at 50% relative humidity and raised on a 12-h light/dark cycle with access to food and water *ad libitum* in the animal facility at Tianjin Medical University. All animal experiments were performed according to the standards in the *Guide for the Care and Use of Laboratory Animals* (U.S. Institute of Laboratory Animal Resources of National Research Council). All experiments were approved by Animal Care and Use Committee at Tianjin Medical University, Tianjin, China.

### Mouse model of acute pyelonephritis.

Acute pyelonephritis in female C57BL/6J mice was induced by kidney inoculation with UPEC via the urethra as previously described (20). UPEC strains were cultured overnight under static conditions in LB medium, harvested by centrifugation at 8,000 × *g* for 5 min, and resuspended in PBS. Anesthetized female C57BL/6J mice were inoculated intraurethrally with 50-μL portions of UPEC strains (10^8^ CFU) twice at 3-h intervals. Mice were euthanized at 12, 24, or 48 hpi. Kidneys were aseptically extracted and homogenized in PBS containing 0.025% Triton X-100. The mixtures were then serially diluted and plated on LB agar plates for bacterial enumeration. Kidneys were also collected for flow cytometry, histopathology, or proinflammatory cytokine level analysis.

### Statistical analysis.

Data are presented as means ± the standard errors of the mean (SEM). The statistical significance of the differences between groups was calculated using a Student *t* test, one-way analysis of variance (ANOVA) with the Tukey *post hoc* test, or two-way ANOVA with the Sidak *post hoc* test. Statistical analyses were performed using Prism 6 (GraphPad, San Diego, CA). *P < *0.05 was considered a statistically significant difference.

### Data availability.

The RNA-seq data have been deposited in NCBI’s Gene Expression Omnibus (GEO) under accession number GSE184193. Other materials and methods in this study are supplied in supplemental material (see TEXT S1).

10.1128/mbio.01147-22.10 TEXT S1 Supplementary materials and methods. Download Text S1, PDF file, 0.3 MB. Copyright © 2022 Sun et al. 2022 Sun et al. https://creativecommons.org/licenses/by/4.0/ This content is distributed under the terms of the Creative Commons Attribution 4.0 International license.
