# Supplementary figures and images for: Interactions of Bacterial Toxin CNF1 and Host JAK1/2 Driven by Liquid-Liquid Phase Separation Enhance Macrophage Polarization
Source: mBio. 2022 Jun 29;13(4):e01147-22. doi: 10.1128/mbio.01147-22 (PMC9426534; doi:10.1128/mbio.01147-22)

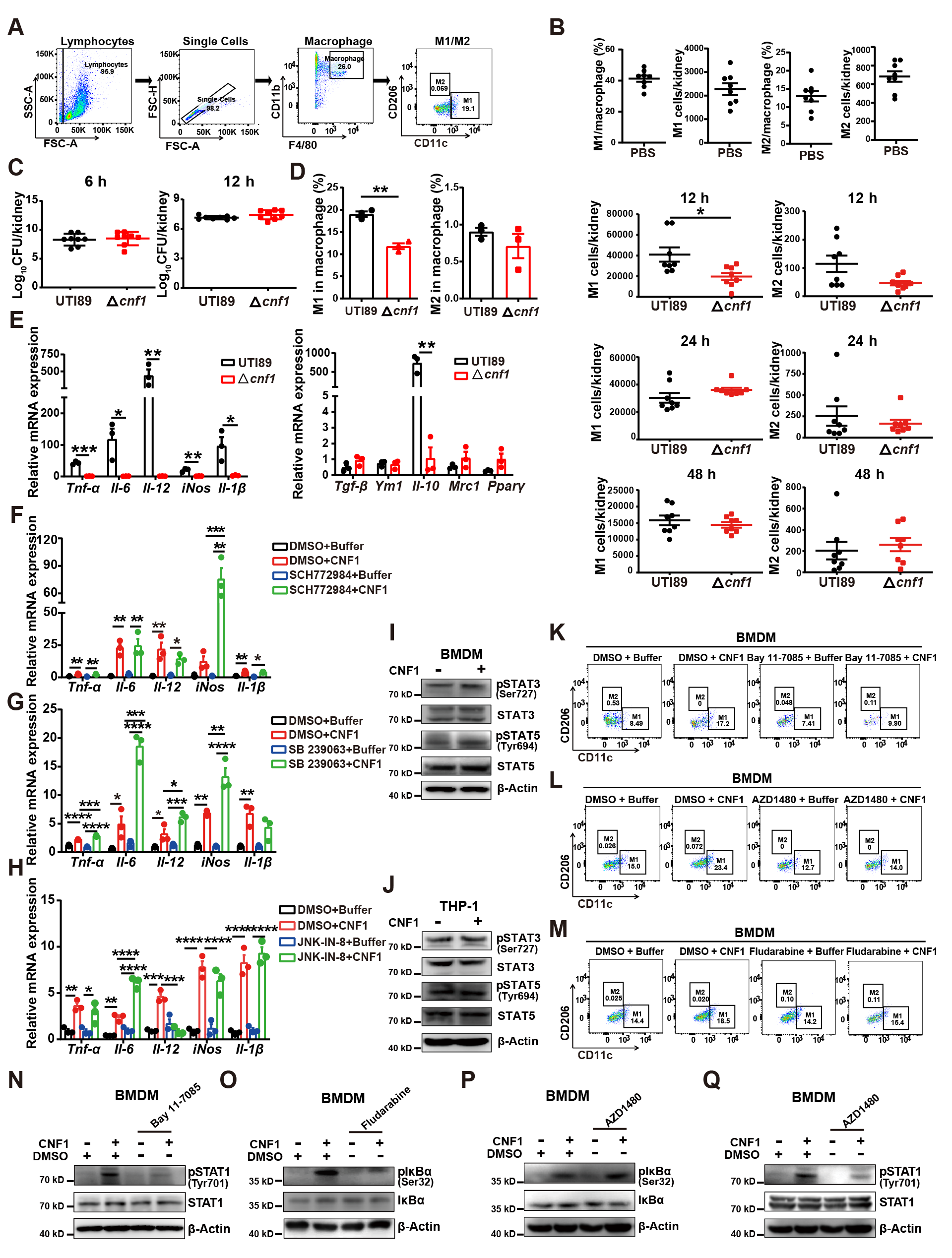

Supplement: FIG S1 [file mbio.01147-22-s0002.tif]

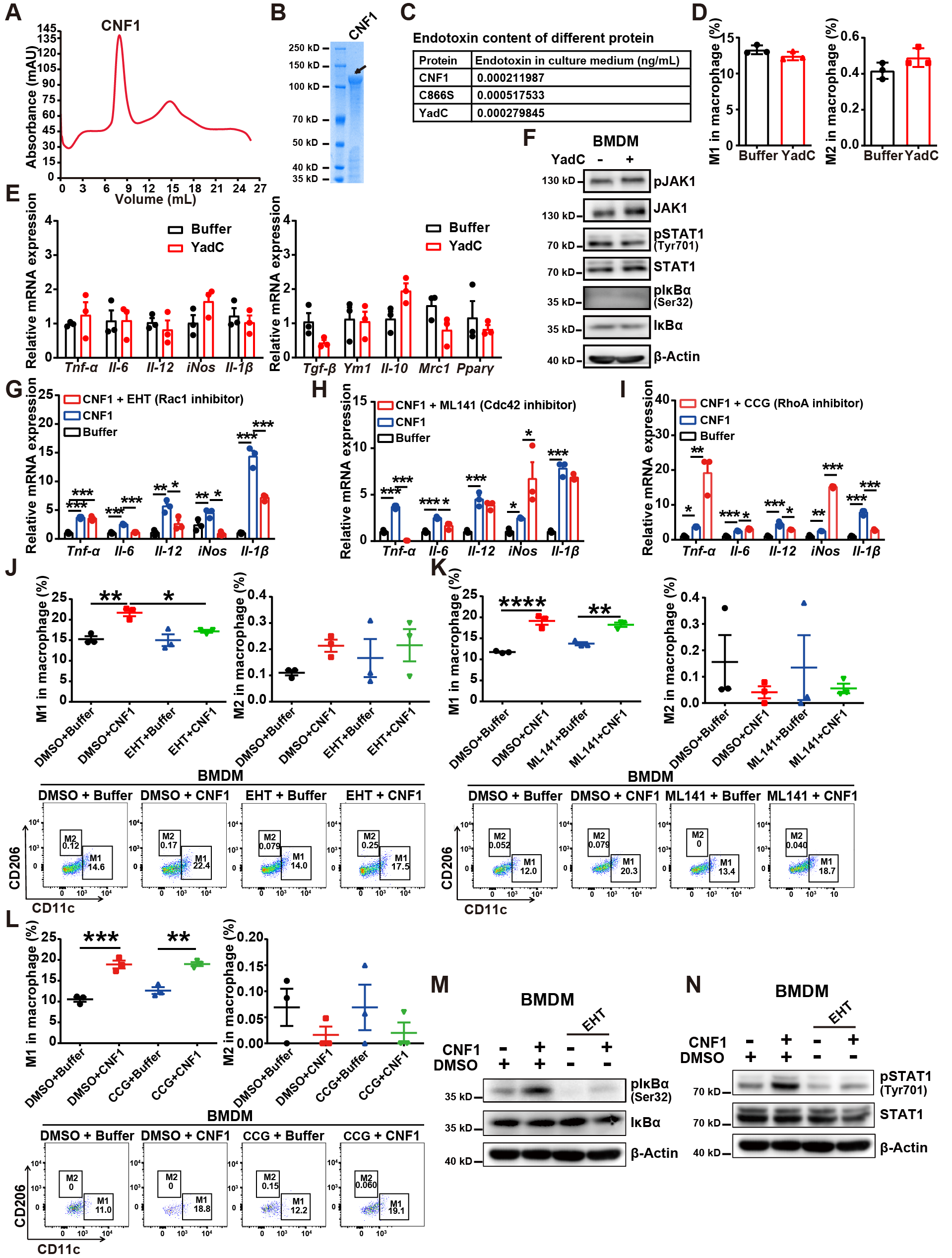

Supplement: FIG S2 [file mbio.01147-22-s0003.tif]

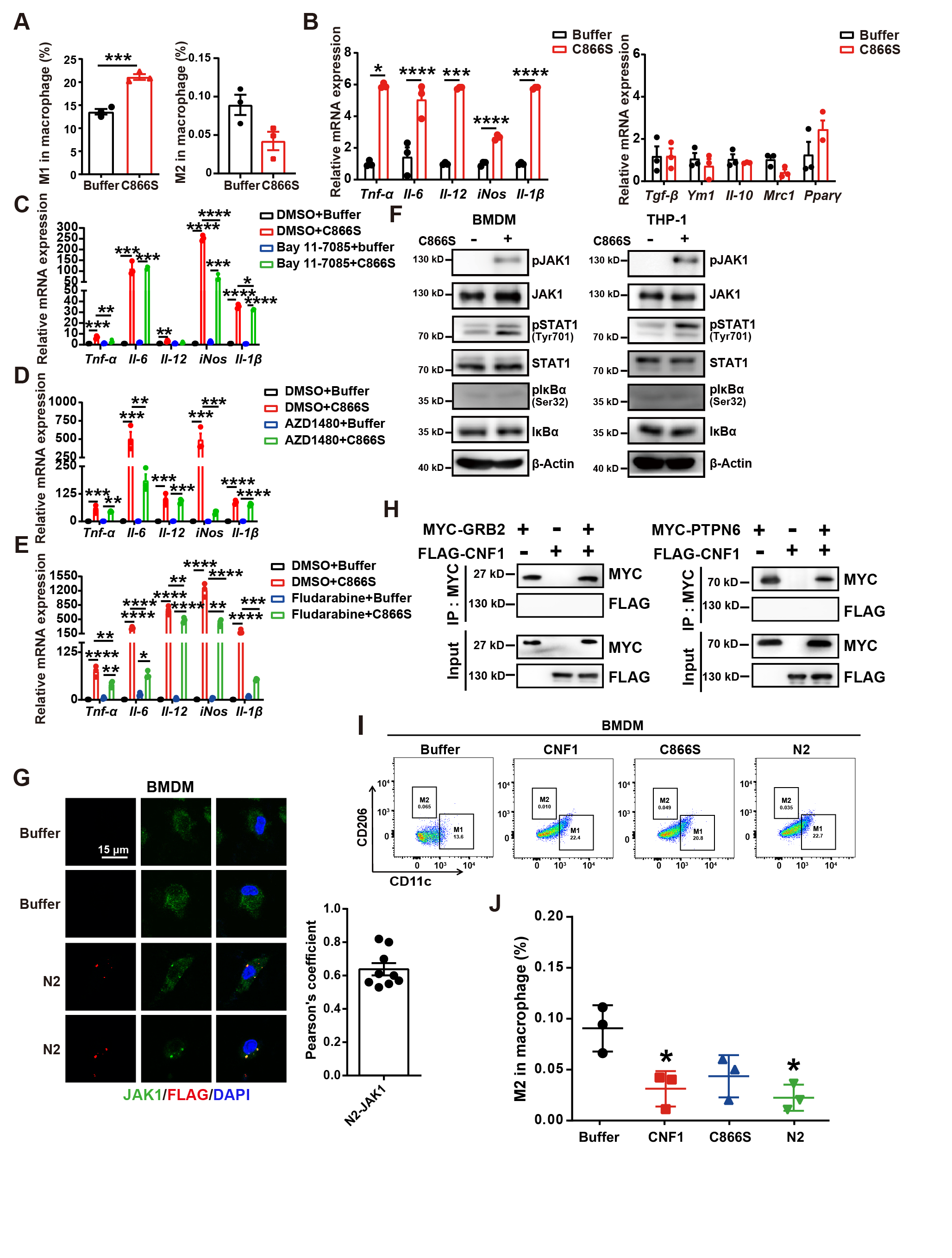

Supplement: FIG S3 [file mbio.01147-22-s0004.tif]

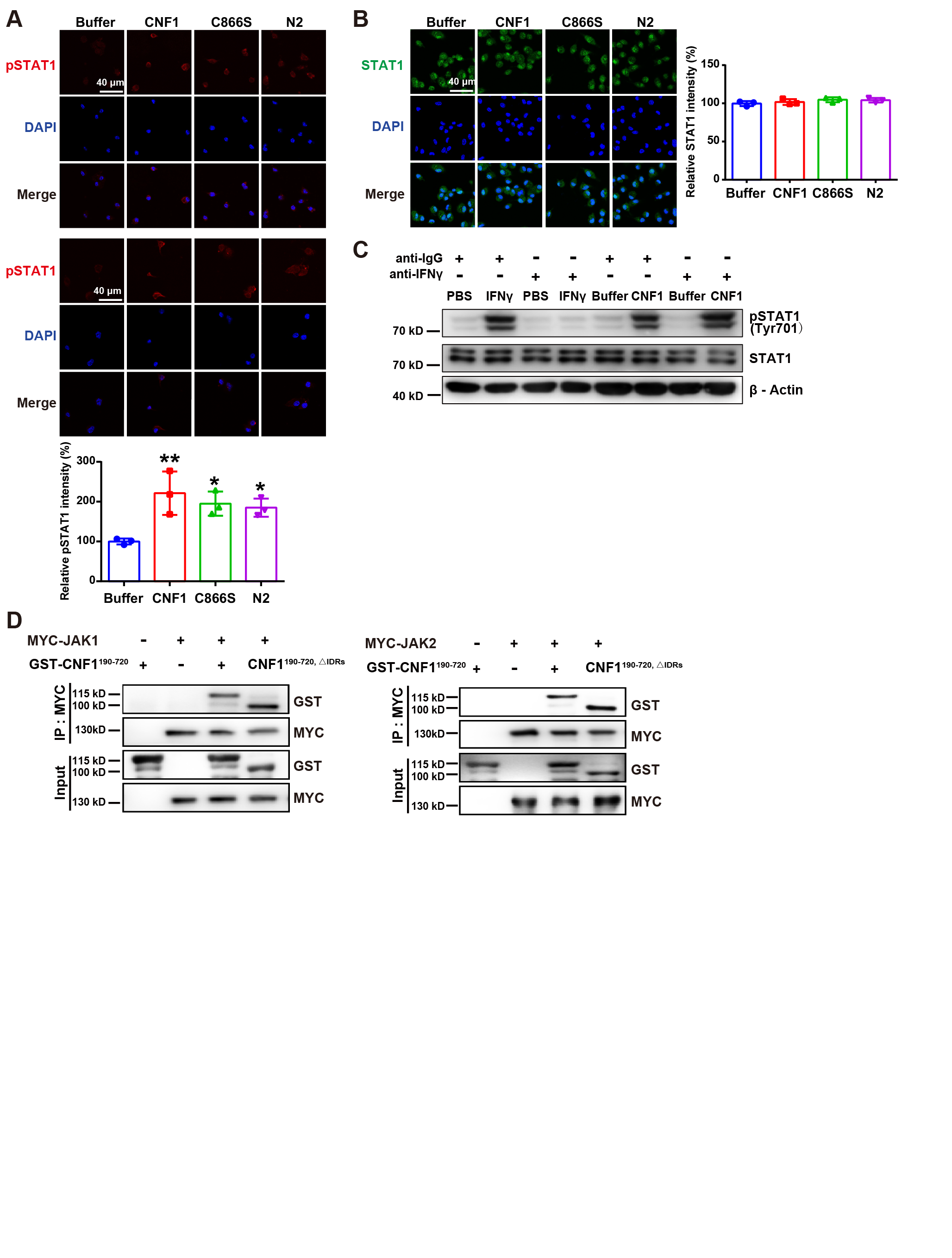

Supplement: FIG S4 [file mbio.01147-22-s0005.tif]

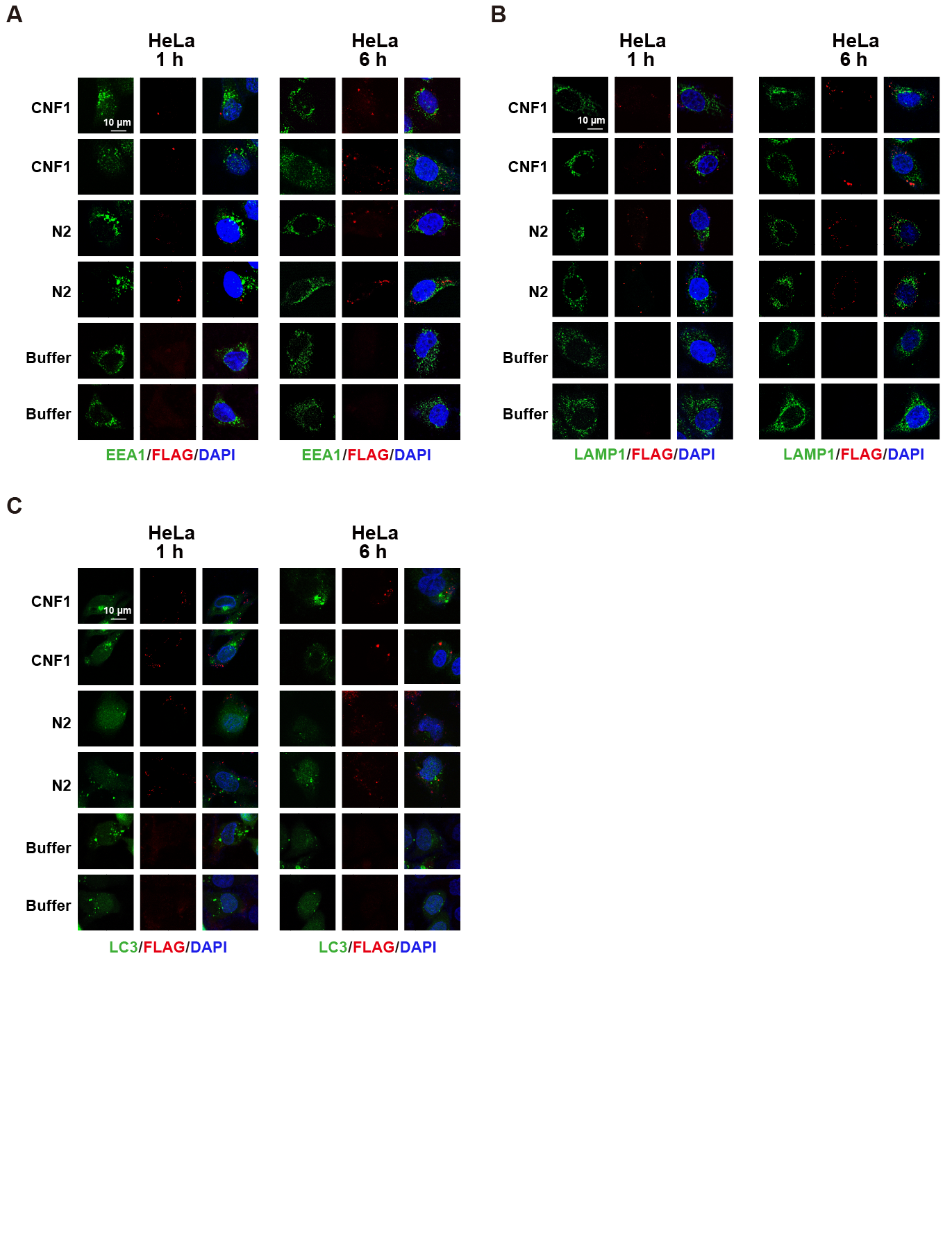

Supplement: FIG S5 [file mbio.01147-22-s0006.tif]

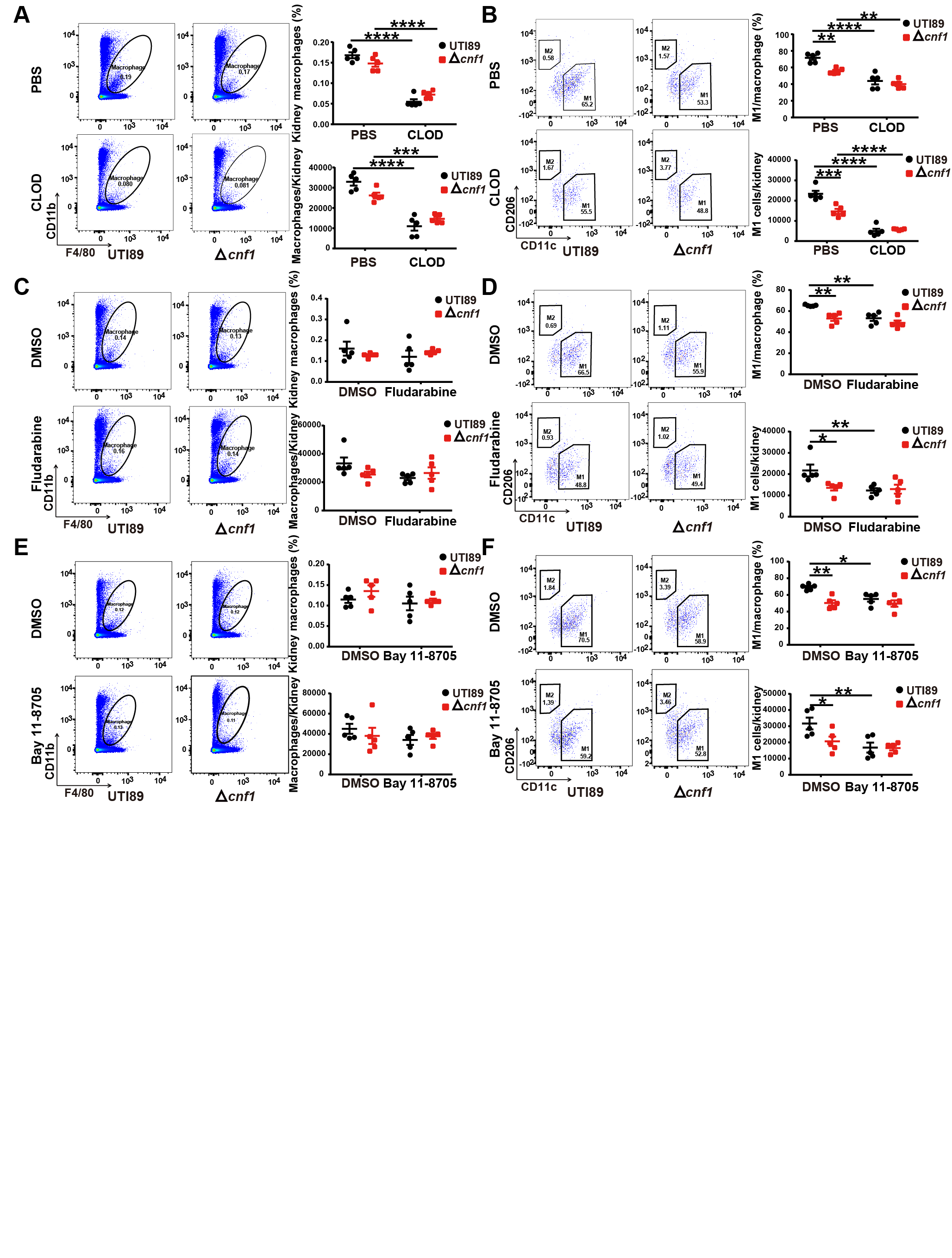

Supplement: FIG S6 [file mbio.01147-22-s0007.tif]
