# Supplementary material for: Interactions of Bacterial Toxin CNF1 and Host JAK1/2 Driven by Liquid-Liquid Phase Separation Enhance Macrophage Polarization
Source: mBio. 2022 Jun 29;13(4):e01147-22. doi: 10.1128/mbio.01147-22 (PMC9426534; doi:10.1128/mbio.01147-22)
Supplement: TABLE S1 [file mbio.01147-22-s0008.pdf]

Table S1. Materials used in this study

REAGENT or RESOURCE	SOURCE	IDENTIFIER
**Antibodies**		
Anti-mouse FLAG	Sigma-Aldrich	Cat# F3165; RRID:AB_259529
Anti-mouse β-Actin	Sigma-Aldrich	Cat# A1978; RRID:AB_476692
Anti-rabbit JAK1	Cell Signaling Technology	Cat# 3344; RRID:AB_2265054
Anti-rabbit pJAK1 (Tyr1034/1035)	Cell Signaling Technology	Cat# 3331; RRID:AB_2265057
Anti-rabbit JAK2	Cell Signaling Technology	Cat# 3230; RRID:AB_2128522
Anti-rabbit pJAK2 (Tyr1007)	Cell Signaling Technology	Cat# 4406; RRID:AB_10706164
Anti-rabbit HA	Cell Signaling Technology	Cat# 3724; RRID:AB_1549585
Anti-rabbit IκBα	Cell Signaling Technology	Cat# 9242; RRID:AB_331623
Anti-rabbit pIκBα (Ser32)	Cell Signaling Technology	Cat# 2859; RRID:AB_561111
Anti-rabbit pSTAT1 (Tyr701)	Cell Signaling Technology	Cat#7649; RRID:AB_10950970
Anti-rabbit pSTAT1 (Tyr701)	Cell Signaling Technology	Cat# 9167 RRID:AB_561284
Anti-rabbit pSTAT3 (Tyr705)	Cell Signaling Technology	Cat# 9145; RRID:AB_2491009
Anti-rabbit pSTAT5 (Tyr694)	Cell Signaling Technology	Cat# 4322; RRID:AB_10544692
Anti-rabbit STAT1	Cell Signaling Technology	Cat# 9172; RRID:AB_2198300
Anti-rabbit STAT3	Cell Signaling Technology	Cat# 12640; RRID:AB_2629499
Anti-rabbit STAT5	Cell Signaling Technology	Cat# 25656; RRID:AB_2798908
Anti-mouse MYC antibody	Proteintech	Cat# 66004-1-Ig; RRID:AB_2881489
Anti-rabbit pIκBα (Ser32/Ser36)	Affinity Biosciences	Cat# AF2002 RRID:AB_2834433
Anti-rabbit F4/80 antibody		Cat# 28463-1-AP RRID:AB_2881149
Anti-rabbit EEA1 antibody	Abcam	Cat# ab109110

		RRID:AB_10863524
Anti-rabbit LAMP1 antibody	Abcam	Cat# ab208943 RRID:AB_2134489
CoraLite 594 conjugated Goat Anti-mouse	Proteintech	Cat# SA00013-3; RRID:AB_2797133
Goat Anti-rabbit	Proteintech	Cat# SA00013-4; RRID:AB_2810984
Anti-mouse CD11b- APC	Thermo Fisher Scientific	Cat# 17-0112-82; RRID:AB_469343
Anti-mouse F4/80-FITC	Thermo Fisher Scientific	Cat# 11-4801-85; RRID:AB_2637192
Anti-mouse CD16/32	Biolegend	Cat# 101319; RRID:AB_1574973
Anti-mouse CD11c-PE	Biolegend	Cat# 117308; RRID:AB_313777
Anti-mouse CD206-PerCP/Cy5.5	Biolegend	Cat# 141716; RRID:AB_2561992
InVivoMab anti-mouse IFNγ	BioXcell	Cat# BE0055; RRID:AB_1107694
InVivoMab mouse IgG1 isotype control	BioXcell	Cat# BE0083; RRID:AB_ 1107757
**Bacterial strains and plasmid**		
E. coli BL21	Merck-Millipoe	Cat# 203790
E. coli DH5α	Lab stored	N/A
pET-28a (+)	Lab stored	N/A
pET-28a (+)-3×FLAG-CNF1	This paper	N/A
pET-28a (+)-3×FLAG-N1	This paper	N/A
pET-28a (+)-3×FLAG-N2	This paper	N/A
pET-28a (+)-3×FLAG-C1	This paper	N/A
pET-28a (+)-3×FLAG-C2	This paper	N/A
pLVX-IRES-Hyg	Lab stored	N/A
pLVX-IRES-Hyg-6×MYC-JAK1	This paper	N/A
pLVX-IRES-Hyg-6×MYC-JAK1 1-420	This paper	N/A
pLVX-IRES-Hyg-6×MYC-JAK1 1-544	This paper	N/A
pLVX-IRES-Hyg-6×MYC-JAK1 1-855	This paper	N/A
pLVX-IRES-Hyg-6×MYC-JAK1 420-1154	This paper	N/A

pLVX-IRES-Hyg-6×MYC-JAK1 544-1154	This paper	N/A
pLVX-IRES-Hyg-6×MYC-JAK1 855-1154	This paper	N/A
pLVX-IRES-Hyg-6×MYC-JAK2	This paper	N/A
pLVX-EF1α-IRES-Puro	Lab stored	N/A
pLVX-EF1α-IRES-Puro-HA-JAK1	This paper	N/A
pLVX-EF1α-IRES-Puro-HA-JAK2	This paper	N/A
psPAX2	Lab stored	N/A
pMD2.G	Lab stored	N/A
pGEX6P1-mCherry	Professor Yupeng Chen's lab	N/A
pGEX6P1-mCherry-JAK1	This paper	N/A
pGEX6P1-mCherry-CNF1	This paper	N/A
pGEX6P1-mCherry-N1	This paper	N/A
pGEX6P1-mCherry-N2	This paper	N/A
pGEX6P1-mCherry-CNF1$^{190\text{-}720,\ IDRs}$	This paper	N/A
pGEX6P1-mCherry-CNF1^{IDRs}	This paper	N/A
pGEX6P1-GFP	Professor Yupeng Chen's lab	N/A
pGEX6P1-GFP-CNF1	This paper	N/A
pGEX6P1-GFP-N1	This paper	N/A
pGEX6P1-GFP-N2	This paper	N/A
pcDNA3-GFP	Professor Yupeng Chen' s lab	N/A
pcDNA3-GFP-JAK1	This paper	N/A
pmCherry-C1	Professor Yupeng Chen's lab	N/A
pmCherry-C1-JAK2	This paper	N/A
**Chemicals, Peptides, and Recombinant Proteins**		
Ampicillin sodium	Sangon Biotech	Cat# 69-52-3
Kanamycin sulfate	Sangon Biotech	Cat#25389-94-0
PBS	Solarbio	Cat# P1020
DMSO	Solarbio	Cat# D8372
4% Paraformaldehyde	Solarbio	Cat# P110
Triton X-100	Solarbio	Cat# T8200

DAPI	Solarbio	Cat# C0060
Benzonase	HaiGene	Cat# C2001
FastDigest NotI	Thermo Fisher Scientific	Cat#FD0594
FastDigest KpnI	Thermo Fisher Scientific	Cat#FD0524
FastDigest SmaI	Thermo Fisher Scientific	Cat#FD0663
Protein A/G agarose	Thermo Fisher Scientific	Cat#20241
HRV 3C Protease	Thermo Fisher Scientific	Cat#88946
Polyethylene glycol, molecular weight 8000	Sigma-Aldrich	Cat#P5413
1, 6-Hexanediol	Sigma-Aldrich	Cat#H11807
Phorbol 12-myristate 13-acetate	Sigma-Aldrich	Cat#P8139
Lysozyme	Sigma-Aldrich	Cat#L6876
DNaseI	New England Biolabs	Cat#M0303
ProteinShow-G250 Protein Stain Reagent	CWBio	Cat#CW0023S
T4 DNA Ligase	New England Biolabs	Cat#M0202S
Q5 High-Fidelity DNA Polymerase	New England Biolabs	Cat#M0491V
3× FLAG Peptide	Sigma-Aldrich	Cat#F4799
Anti-c-Myc Agarose Affinity Gel antibody produced in rabbit	Sigma-Aldrich	Cat#A7470
c-Myc Peptides	Sigma-Aldrich	Cat#M2435
Anti-FLAG(R) M2 Affinity Gel	Sigma-Aldrich	Cat# A2220
Collagenase IV	Sigma-Aldrich	Cat# C5138
Ni-NTA Purification System	GenScript	Cat#L00250
Glutathione Sepharose 4B	Cytiva	Cat#17075601
Complete mini-EDTA-free protease inhibitor cocktail tablets	Roche	Cat#11697498001
Kinase buffer	Cell Signaling Technology	Cat#9802S
ATP	Cell Signaling Technology	Cat#9804S
Clodronate liposomes	Target Technology	Cat#CP-005-005
Bay 11-7085	MedChem	Cat#HY-10257

	Express	
Fludarabine	MedChem Express	Cat#HY-10257
AZD-1480	MedChem Express	Cat#HY-10193
CCG-1423	MedChem Express	Cat#HY-13991
ML141	MedChem Express	Cat#HY-12755
EHT 1864	MedChem Express	Cat#HY-16659
SB 203580	MedChem Express	Cat#HY-10256
JNK-IN-8	MedChem Express	Cat#HY-13319
SCH772984	MedChem Express	Cat#HY-13319
Murine macrophage colony-stimulating factor	PeproTech	Cat# 315-02-50UG
**Critical Commercial Assays**		
HiFiScript cDNA Synthesis Kit	CWBio	Cat# CW2569M
Ultra SYBR Mixture	CWBio	Cat# CW0957M
Mouse TNFα ELISA kit	Neobioscience	Cat#EMC102a.96
Mouse IL-12 ELISA kit	Neobioscience	Cat#EMC006.96
Mouse IL-1β ELISA kit	Neobioscience	Cat#EMC001b.96
Mouse IL-6 ELISA kit	Neobioscience	Cat#EMC004.96
Silver staining kit	Thermo Fisher Scientific	Cat#24612
BCA Protein Assay Kit	Thermo Fisher Scientific	Cat#23225
Total RNA Extraction Kit	Solarbio	Cat#R1200
EndoFree Maxi Plasmid Kit	TIANGEN	Cat#DP117
Gel Extraction Kit	CWBio	Cat#CW2302M
EndoFree Plasmid Midi Kit	CWBio	Cat#CW2105S
**Deposited Data**		
RNA-Seq	This paper	GEO: GSE184193
**Experimental Models: Organisms/Strains**		
C57BL/6J mouse	Academy of Military Medical Science (China)	N/A

Uropathogenic E. coli strain (UTI89)	Professor Harry Mobley's lab	N/A
cnf1 deletion strain derived from UTI89 (Δcnf1)	Lab stored	N/A
**Oligonucleotides**		
β-actin F: GGCTGTATTCCCCTCCATCG	Sangon Biotech	Custom made
β-actin R: CCAGTTGGTAACAATGCCATGT	Sangon Biotech	Custom made
Pparg F: TCGCTGATGCACTGCCTATG	Sangon Biotech	Custom made
Pparg R: GAGAGGTCCACAGAGCTGATT	Sangon Biotech	Custom made
Ym-1 F: CAGGTCTGGCAATTCTTCTGAA	Sangon Biotech	Custom made
Ym-1 R: GTCTTGCTCATGTGTGTAAGTGA	Sangon Biotech	Custom made
Arg1 F: TCACCTGAGCTTTGATGTCG	Sangon Biotech	Custom made
Arg1 R: CTGAAAGGAGCCCTGTCTTG	Sangon Biotech	Custom made
IL-10 F: CACCTACTTCCCAGCCAACC	Sangon Biotech	Custom made
IL-10 R: TCAGCAGAGACTCACTCAGCAAC	Sangon Biotech	Custom made
TGF-β F: CAATTCCTGGCGATACCTCAG	Sangon Biotech	Custom made
TGF-β R: GCACAACTCCGGTGACATCAA	Sangon Biotech	Custom made
IL-6 F: TAGTCCTTCCTACCCCAATTTCC	Sangon Biotech	Custom made
IL-6 R: TTGGTCCTTAGCCACTCCTTC	Sangon Biotech	Custom made
iNos F: GTTCTCAGCCCAACAATACAAGA	Sangon Biotech	Custom made
iNos R: GTGGACGGGTCGATGTCAC	Sangon Biotech	Custom made
TNFa F: CCCTCACACTCAGATCATCTTCT	Sangon Biotech	Custom made
TNFa R: GCTACGACGTGGGCTACAG	Sangon Biotech	Custom made
IL-12 F: TGGTTTGCCATCGTTTTGCTG	Sangon Biotech	Custom made
IL-12 R: ACAGGTGAGGTTCACTGTTTCT	Sangon Biotech	Custom made
IL-1β F: GCAACTGTTCCTGAACTCAACT	Sangon Biotech	Custom made
IL-1β R: ATCTTTTGGGGTCCGTCAACT	Sangon Biotech	Custom made
IL-1β R: ATCTTTTGGGGTCCGTCAACT	Sangon Biotech	Custom made
Cloning CNF1, N1 and N2 into PET-28a(+)-3xFLAG F: CGGGATCCATGGGTAACCAATGGCAACAAAAA	Sangon Biotech	Custom made
Cloning C2 into pET-28a(+)-3xFLAG F: CGGGATCCATGAGTATCGAAAGCACCTCCAAA	Sangon Biotech	Custom made
Cloning C1 into pET-28a(+)-3×FLAG F: CGGGATCCATGCGCAGTGATAATATATCACTA	Sangon Biotech	Custom made
Cloning CNF1, C1 and C2 into PET-28a(+)-3×FLAG R: ATAAGAATGCGGCCGCTCTAGATTTGTCGTCATCA TC	Sangon Biotech	Custom made
Cloning N1 into pET-28a(+)-3×FLAG R: CGGGATCCCTGCGATTTAAGGCGTTCATGGA	Sangon Biotech	Custom made

Cloning N2 into pET-28a(+)-3×FLAG R: CGGGATCCATACTTTCTTTAAAATATCGTAG	Sangon Biotech	Custom made
Cloning JAK1 855-1154 into pLVX-IRES-Hyg-6×MYC F: CCGCTCGAGATGATTGTTTCAGAAAAAAAACCA	Sangon Biotech	Custom made
Cloning JAK1 544-1154 into pLVX-IRES-Hyg-6×MYC F: CCGCTCGAGATGTGCCAGCCCAAGCCCCGAGAA	Sangon Biotech	Custom made
Cloning JAK1 420-1154 into pLVX-IRES-Hyg-6×MYC F: CCGCTCGAGATGCATCATTACCTCTGCACCGACGT	Sangon Biotech	Custom made
Cloning JAK1, JAK1 1-420, JAK1 1-544 and JAK1 1-855 into pLVX-IRES-Hyg-6×MYC F: CCGCTCGAGCAGTATCTAAATATAAAAGA	Sangon Biotech	Custom made
Cloning JAK1, JAK1 420-1154, JAK1 544-1154 and JAK1 855-1154 into pLVX-IRES-Hyg-6×MYC R: ATAAGAATGCGGCCGCTTATTTTAAAAGTGCTTCAAATC	Sangon Biotech	Custom made
Cloning JAK1 1-420 into pLVX-IRES-Hyg-6×MYC R: ATAAGAATGCGGCCGCTTAATGGGCATCTGCTGTGAGC	Sangon Biotech	Custom made
Cloning JAK1 1-544 into pLVX-IRES-Hyg-6×MYC R: ATAAGAATGCGGCCGCTTAGCAGCAGCGTTTTAGCATGAA	Sangon Biotech	Custom made
Cloning JAK1 1-855 into pLVX-IRES-Hyg-6×MYC R: ATAAGAATGCGGCCGCTTAAATATCTGGATTCTGCTCTTC	Sangon Biotech	Custom made
Cloning JAK2 into pLVX-IRES-Hyg-6×MYC F: AATTCCTCGAGACTAGTTCTAGAATGGGAATGGCCTGCCTTACG	Sangon Biotech	Custom made
Cloning JAK2 into pLVX-IRES-Hyg-6×MYC R: GGAGGGAGAGGGGCGGGATCCTCATCCAGCCATGTTATCCCT	Sangon Biotech	Custom made
Cloning JAK1 into pLVX-EF1α-IRES-Puro-HA F: ATGTACCCATACGACGTCCCAGACTACGCTCAGTATCTAAATATAAAAGA	Sangon Biotech	Custom made
Cloning JAK1 into pLVX-EF1α-IRES-Puro-HA F: AATTCCTCGAGACTAGTTCTAGAATGTACCCATACGACGTCCCAGAC	Sangon Biotech	Custom made
Cloning JAK1 into pLVX-EF1α-IRES-Puro-HA R: GGAGGGAGAGGGGCGGGATCCTTATTTTAAAAGTGCTTCAAATC	Sangon Biotech	Custom made
Cloning JAK2 into pLVX-EF1α-IRES-Puro-HA F: ATGTACCCATACGACGTCCCAGACTACGCTGGAATGGCCTGCCTTACGAT	Sangon Biotech	Custom made

Cloning JAK2 into pLVX-EF1α-IRES-Puro-HA F: AATTCCTCGAGACTAGTTCTAGAATGTACCCATAC GACGTCCCAGAC	Sangon Biotech	Custom made
Cloning JAK2 into pLVX-EF1α-IRES-Puro-HA R: GGAGGGAGAGGGGCGGGATCCTCATCCAGCCATG TTATCCCTTA	Sangon Biotech	Custom made
Cloning CNF1, CNF1^{IDRs}， CNF1 1-190 and CNF1 1-720 into pGEX6P1-mCherry F: CTGTTCCAGGGGCCCCTGGGATCCATGGGTAACCA ATGGCAACAAAAA	Sangon Biotech	Custom made
Cloning CNF1, and CNF1^{IDRs} into pGEX6P1-mCherry RCTCGAGTCGACCCGGGAATTCAAATTTTTTTGAA AATACCTTCAA	Sangon Biotech	Custom made
Cloning N1 into pGEX6P1-mCherry R: CTCGAGTCGACCCGGGAATTCGCGATTTAAGGCGT TCATGGA	Sangon Biotech	Custom made
Cloning N2 into pGEX6P1-mCherry R: CTCGAGTCGACCCGGGAATTCACTTTCTTTAAAAT ATCGTAG	Sangon Biotech	Custom made
Cloning JAK1 into pGEX6P1-mCherry F: CTGTTCCAGGGGCCCCTGGGATCCATGCAGTATCT AAATATAAAAGA	Sangon Biotech	Custom made
Cloning JAK1 into pGEX6P1-mCherry R: CTCGAGTCGACCCGGGAATTCTTTTAAAAGTGCTT CAAATC	Sangon Biotech	Custom made
Cloning JAK1 into pcDNA3-GFP F: CGGGGTACCATGCAGTATCTAAATATAAAAGA	Sangon Biotech	Custom made
Cloning JAK1 into pcDNA3-GFP R: CCGCTCGAGTTTTAAAAGTGCTTCAAATC	Sangon Biotech	Custom made
Cloning JAK2 into pmCherry-C1 F: CGAGCTCTCGGAATGGCCTGCCTTACGAT	Sangon Biotech	Custom made
Cloning JAK2 into pmCherry-C1 R: ACGCGTCGACTCATCCAGCCATGTTATCCCTTA	Sangon Biotech	Custom made
Cloning CNF1 and CNF1^{IDRs} into pGEX6P1-mCherry R CTCGAGTCGACCCGGGAATTCAAATTTTTTTGAAA ATACCTTCAA	Sangon Biotech	Custom made
Cloning CNF1 1-190 into pGEX6P1-mCherry R CTCGAGTCGACCCGGGAATTCGCGATTTAAGGCGT TCATGGA	Sangon Biotech	Custom made
Cloning CNF1 1-720 into pGEX6P1-mCherry R CTCGAGTCGACCCGGGAATTCACTTTCTTTAAAAT ATCGTAG	Sangon Biotech	Custom made

Cloning CNF1, CNF1 1-190 and CNF1 1-720 into pGEX6P1-GFP F CTGTTCCAGGGGCCCCTGGGATCCATGGGTAACCA ATGGCAACAAAAA	Sangon Biotech	Custom made
Cloning CNF1 into pGEX6P1-GFP R CTCCTCGCCCTTGCTCACCATGAATTCAAATTTTTT TGAAAATACCTTCAA	Sangon Biotech	Custom made
Cloning CNF1 1-190 into pGEX6P1-GFP R CTCCTCGCCCTTGCTCACCATGAATTCGCGATTTA AGGCGTTCATGGA	Sangon Biotech	Custom made
Cloning CNF1 1-720 into pGEX6P1-GFP R CTCCTCGCCCTTGCTCACCATGAATTCACTTTCTTT AAAATATCGTAG	Sangon Biotech	Custom made
Cloning CNF1 190-720 and CNF1$^{190-720, IDRs}$ into pGEX6P1-mCherry F CTGTTCCAGGGGCCCCTGGGATCCATGCGCAGTGA TAATATATCACTA	Sangon Biotech	Custom made
Cloning CNF1 190-720 and CNF1$^{190-720, IDRs}$ into pGEX6P1-mCherry R CTCGAGTCGACCCGGGAATTCACTTTCTTTAAAAT ATCGTAG	Sangon Biotech	Custom made
Cloning CNF1^{IDRs} into pGEX6P1-mCherry F ATATTTTTGTAGCCAAAAATTATAGTCGTCAAGAT GGAGT	Sangon Biotech	Custom made
Cloning CNF1^{IDRs} into pGEX6P1-mCherry R ACTCCATCTTGACGACTATAATTTTTGGCTACAAA AATAT	Sangon Biotech	Custom made
Cloning CNF1^{IDRs} into pGEX6P1-mCherry F TTGCAATTCCACTCTTGATTAAAGGGAGTTCTCTG AGCGG	Sangon Biotech	Custom made
Cloning CNF1^{IDRs} into pGEX6P1-mCherry F CCGCTCAGAGAACTCCCTTTAATCAAGAGTGGAAT TGCAA	Sangon Biotech	Custom made
**Software and Algorithms**		
FlowJo10.4	TreeStar	https://www.flowjo.com RRID:SCR_008520
Prism 6	GraphPad software	https://www.graphpad.com/ RRID:SCR_002798
ImageJ	ImageJ	https://www.imagej.net/RRID:SCR_003070
Image-Pro Plus 6	Image-Pro Plus	https://www.mediacy.com/imageproplus/ RRID: SCR_016879
