# Supplementary material for: Interactions of Bacterial Toxin CNF1 and Host JAK1/2 Driven by Liquid-Liquid Phase Separation Enhance Macrophage Polarization
Source: mBio. 2022 Jun 29;13(4):e01147-22. doi: 10.1128/mbio.01147-22 (PMC9426534; doi:10.1128/mbio.01147-22)
Supplement: TEXT S1 [file mbio.01147-22-s0001.pdf]

Supplementary materials and methods

Cell lines. Human embryonic kidney 293T cells (ATCC CRL-3216), human cervical cancer HeLa cells (ATCC CCL-2) and human myeloid leukemia mononuclear THP-1 cells (ATCC TIB-202) was obtained from ATCC. 293T and HeLa cells were grown in Dulbecco's modified Eagle's medium (DMEM) supplemented with 10% fetal bovine serum. THP-1 cells were cultured in RPMI 1640 medium supplemented with 10% fetal bovine serum. Cells were incubated at 37 °C in a humidified incubator containing 5% CO_2.

THP-1-derived macrophages. THP-1 cells were re-suspended in RPMI 1640 medium supplemented with 10% fetal bovine serum and were differentiated into macrophages by treatment with 50 ng/ml phorbol 12-myristate 13-acetate (P8139, Sigma-Aldrich, St. Louis, MO, USA) for 24 h.

Bone marrow derived macrophages. Bone marrow derived macrophages (BMDMs) were isolated from female C57BL/6J mice, and cultured in Dulbecco's modified Eagle's medium/F12 containing 10% heat inactivated fetal bovine serum, penicillin (100 U/ml), streptomycin (100 μg/ml), and 10 ng/ml of murine M-CSF (315-02-50, Peprotech, Cranbury, NJ, USA). Cells were differentiated for 6 days in the presence of M-CSF. The culture medium was refreshed every 3 days.

Recombinant protein expression and purification. BL21 *E. coli* competent bacteria were transformed with the overexpression plasmids pET-28a (+)-3×FLAG-CNF1, pET-28a (+)-3×FLAG-N1, pET-28a (+)-3×FLAG-N2, pET-28a (+)-3×FLAG-C1 or pET-28a (+)-3×FLAG-C2, pET-28a (+)-FLAG-YadC, pGEX6P1-GFP-CNF1, pGEX6P1-GFP-N1, pGEX6P1-GFP-N2, pGEX6P1-GFP, pGEX6P1-mCherry-CNF1, pGEX6P1-mCherry-N1, pGEX6P1-mCherry-N2, pGEX6P1-mCherry-CNF1$^{190\text{-}720}$, pGEX6P1-mCherry-CNF1$^{190\text{-}720,\ \triangle\ \mathrm{IDRs}}$, and pGEX6P1-mCherry, and pGEX6P1-

mCherry-JAK1. The bacteria were grown in LB broth at 37 °C to an OD600 of 0.6 to 0.8. FLAG-tagged proteins, GST-tagged proteins, GFP fusion proteins, and mCherry fusion proteins were expressed by induction of 100 μM isopropyl β-D-1-thiogalactopyranoside (IPTG, B300845, Sangon Biotech, Shanghai, China) at 16 °C for 18 h. Cultured bacteria were harvested through centrifugation at 8000×g for 5 min at 4 °C. The harvested cells were re-suspended in lysis buffer (50 mM Tris-HCl pH 7.5, 100 mM NaCl, and 10 μg/μl Lysozyme (L6876, Sigma-Aldrich)) supplemented with complete EDTA-free protease inhibitor cocktail (11697498001, Roche, Indianapolis, IN) and the protein-containing supernatants were collected after centrifugation at 8,000×g for 30 min at 4 °C. Purification and elution of FLAG-tagged proteins were performed by the Ni-NTA Purification System (L00250, GenScript, Nanjing, China) according to the instructions. After dialysis against PBS, FLAG-tagged proteins were concentrated using Amicon Ultra-15 Centrifugal Filter Units (UFC903096, Millipore, Burlington, MA, USA). To eliminate contaminating bacterial endotoxins, the proteins were further purified by size-exclusion chromatography using the AKTA Pure chromatography system (29018224, Cytiva, Marlborough, MA, USA). The endotoxin level was measured using the ToxinsensorTM LAL endotoxin test kit (L00350, Genscript, Piscataway, NJ, USA). Proteins were detected by Coomassie blue staining and concentration was determined spectrophotometrically (Nanodrop-2000, Thermo Scientific, Waltham, MA, USA) using the BCA Protein Assay Kit (23225, Thermo Fisher).

For purification of recombinant mCherry and GFP fusion proteins, bacterial pellets were resuspended in 10 ml of GST lysis buffer (50 mM Tris-HCl pH 7.5, 100 mM NaCl and 1% Triton X-100) containing protease inhibitor cocktail tablets (11697498001, Roche). After sonication and centrifugation, the supernatant was added to Glutathione

Sepharose 4B (17075601, Cytiva) for 12 h at 4°C with constant rotation to combine with mCherry and GFP fusion proteins. After washing with lysis buffer, cleavage buffer (50 mM Tris pH 7.5, 150 mM NaCl) containing HRV 3C Protease (88946, Thermo Scientific) was added to remove the GST affinity tags. For recombinant GST-tagged protein purification, the supernatant was added to Glutathione Sepharose 4B (17075601, Cytiva) with constant rotation to combine with GST-tagged proteins. GST-tagged proteins combined with Glutathione Sepharose 4B (17075601, Cytiva) were washed with lysis buffer and then eluted by eluent（20 mM Tris-HCl, 100 mM NaCl, 200 mM reduced glutathione and 0.5% Triton X-100 pH 7.5). After buffer containing GST-tagged, mCherry, and GFP fusion proteins dialysis against PBS, they were concentrated using Amicon Ultra-15 Centrifugal Filter Units (UFC903096, Millipore). Proteins were detected by Coomassie blue staining and concentration was determined spectrophotometrically (Nanodrop-2000, Thermo Scientific) using the BCA Protein Assay Kit (23225, Thermo Fisher).

For recombinant MYC-tagged protein purification, the recombinant plasmid was successfully overexpressed in 293T cells. Cell pellets were resuspended in lysis buffer (2 mM EDTA, 50 mM Tris-HCl pH 7.4, 150 mM NaCl, 1% NP-40, 20% glycerol) with complete mini-EDTA-free protease inhibitor cocktail tablets (11697498001, Roche). After centrifugation, the protein-containing supernatant was incubated with anti-c-MYC beads (A7470, Sigma) for 12 h at 4 °C with constant rotation. Anti-c-MYC beads were then incubated with c-MYC peptides (M2435, Sigma) in TBS buffer (20 mM Tris-HCl pH 7.6, 140 mM NaCl) on ice for 2 h and centrifuged again to collect the recombinant MYC-tagged proteins. Protein concentration was determined spectrophotometrically (Nanodrop-2000, Thermo Scientific) using the BCA Protein Assay Kit (23225, Thermo Fisher).

Flow cytometry analysis. The BMDMs suspension was collected and washed twice with PBS. For single-cell suspension of kidney, kidneys were sliced into small pieces and digested with 1.5 mg/ml collagenase IV (C5138, Sigma) and 100 ng/ml DNaseI (M0303S, New England Biolabs, Beverly, MA, USA) in PBS for 30 min at 37 °C under mild shaking. The digested cell suspensions were then filtered through a 70 μm cell strainer (352350, BD Biosciences, San Jose, CA, USA) to obtain single-cell suspensions. The single-cell suspensions were blocked by Fc receptors blocking reagent CD16/32 (101319, Biolegend, San Diego, CA, USA) and then were stained with anti-CD11b conjugated to APC (17-0112-82, eBioscience), anti-F4/80 conjugated to FITC (11-4801-85, eBioscience), anti-CD11c conjugated to PE (117308, Biolegend), anti-CD206 conjugated to PerCP/Cy5.5 (141716, Biolegend). Stained cells were analyzed on a FACS Canto II Flow Cytometer (BD Biosciences) using the FlowJo software (FlowJo, Ashland, OR, USA).

DNA constructs. The *cnf1* genes from UPEC and truncation mutants of *cnf1* genes was amplified by PCR and cloned to pET-28a (+) vector (Novagen, Madison, WI, USA) with an C-terminal 3×FLAG tag to produce recombinant FLAG-tagged CNF1 proteins and truncation mutants of recombinant FLAG-tagged CNF1 proteins. Moreover, *cnf1* genes and truncation mutants of *cnf1* genes was amplified by PCR and cloned to pGEX6P1-mCherry or pGEX6P1-GFP vector to produce recombinant GST-tagged CNF1 proteins and truncation mutants of recombinant GST-tagged CNF1 proteins with mCherry or GFP at C-terminus. Human *Jak1* and *Jak2* was amplified by PCR and cloned to pLVX-IRES-Hyg vector (Clontech, Mountain View, CA, USA) with an N-terminal 6×MYC tag, cloned to pLVX-EF1a-IRES-Puro vector with an N-terminal HA tag, or cloned to pcDNA3-GFP/pmCherry-C1 vector with an N-terminal GFP/mCherry tag. Human *Jak1* also was cloned to pGEX6P1-mCherry vector to produce recombinant

GST-tagged JAK1 proteins with mCherry at C-terminus. Moreover, truncation mutants of *Jak1* were amplified by PCR and cloned to pLVX-IRES-Hyg vector with an N-terminal 6×MYC tag. The *yadC* gene from UPEC was amplified by PCR and cloned to pET-28a (+) vector (Novagen) with a C-terminal FLAG tag to produce recombinant FLAG-tagged YadC proteins. Human *grb2* and *ptpn6* was amplified by PCR and cloned to pET-28a (+) vector (Novagen) with an N-terminal MYC tag to produce recombinant MYC-tagged PTPN6 and GRB2 proteins. The constructed plasmids and primers are listed in Table S3.

DNA transfection. The constructed plasmids pLVX-EF1a-IRES-Puro-HA-JAK1, pLVX-EF1a-IRES-Puro-HA-JAK2 or pLVX-IRES-Hyg-6×MYC-JAK1 in addition with assistant vectors psPAX2 and pMD2.G, were transiently transfected into 293T cells. Viral supernatants were collected at 48 h post transfection, clarified by filtration and concentrated by ultracentrifugation. Medium containing virus incubated with targeted HeLa cells for 12 h. Infected HeLa cells were purified by drug selection.

293T cells were transfected with plasmids encoding 6×MYC-JAK1 and truncation mutants of 6×MYC-JAK1 and 6×MYC-JAK2 using polyethylenimine (23966, Polysciences, Warrington, PA, USA) following the manufacturer's instruction. At 48 h post transfection, the cells were collected and confirmed by western blotting with specific antibodies.

HeLa cells were transfected with plasmids encoding JAK1-GFP and JAK2-mCherry using Xfect Transfection Reagent (631317, Clontech, CA, USA) following the manufacturer's instruction. For co-localization analysis between CNF1/N2 and LC3, HeLa cells were transfected with plasmids encoding LC3-EGFP. At 48 h post transfection, the HeLa cells were treated with CNF1/N2 (15 nM) or dialysis buffer for 6h and imaged using a confocal fluorescence microscope (Leica TCS-SP8, Leica

Microsystems).

RNA sequencing. After treatment with CNF1 or dialysis buffer for 6 h, total RNA of BMDMs was extracted using the TRIzol reagent (R1200, Solarbio). Samples were submitted to Shanghai Majorbio Bio-pharm Technology Corporation for RNA sequencing. The data were analyzed on the online platform of Majorbio I-Sanger Cloud Platform (www.isanger.com). The RNA-seq data have been deposited in NCBI's Gene Expression Omnibus (GEO) with the accession number GSE184193.

Enzyme-linked immunosorbent assay (ELISA). The kidneys of mice were extracted and homogenized in PBS containing 1% Triton X-100 and complete mini-EDTA-free protease inhibitor cocktail tablets (11697498001, Roche). The homogenates centrifuged at 12000 × g for 10 min at 4 °C to collect supernatant of homogenized kidneys. TNFα (EMC102a.96, Neobioscience, Shenzhen, China), IL-12 (EMC006, Neobioscience), IL-1β (EMC001b.96, Neobioscience) and IL-6 (EMC004.96, Neobioscience) expression were analyzed using ELISA kits according to the manufacturer's protocols. Plates were read using the Thermo Multiskan Ascent Microplate Reader (Thermo Scientific) and analyte concentrations were calculated with ProcartaPlex Analyst 1.0 Software (Thermo Scientific).

Infection of macrophages with UPEC strains. BMDMs were seeded in 6-well plates or 100 mm diameter culture dishes for 24 h before infection. Cells were infected with UTI89 and Δ*cnf1* strain at MOI of 5 for 6 h. Cells were washed twice with PBS, and then collected for subsequent analysis.

CNF1 and its variants treatment. BMDMs, THP-1, and HeLa cells were seeded in 6-well plates, 100 mm diameter culture dishes, or LAB-TEK 4-well chamber slides (177399, Thermo Scientific) for 24 h. BMDMs and THP-1 cells were treated with purified CNF1 or its variants at 3 nM for 6 h. HeLa cells were treated with purified

CNF1 or its variants at 15 nM for 6 h. Cells were washed twice with PBS, and then collected for subsequent analysis.

Signal transduction inhibitors treatment in BMDMs. BMDMs were pretreated with different inhibitors (2 μM, Bay 11-7085, HY-10257; 50 μM, Fludarabine, HY-B0069; 10 μM, AZD-1480, HY-10193; 20 μM, CCG-1423, HY-13991;10 μM, ML141, HY-12755; 20 μM, EHT 1864, HY-16659;3 μM, SB 203580, HY-10256; 3 μM JNK-IN-8, HY-13319;1 μM, SCH772984, HY-50846) for 2 h. These inhibitors were purchased from MedChem Express (Monmouth Junction, NJ, USA).

Real-time PCR. Total RNA from BMDMs with different treatment was extracted using TRizol reagent (R1200, Solarbio, Beijing, China) in accordance with the manufacturer's instructions, and 2 μg RNA was converted to cDNA using HiFiScript cDNA Synthesis Kit (CW2569M, Cwbio, Beijing, China). Real-time Polymerase Chain Reaction (RT-PCR) was performed using 20 μl reaction mixture contained 20 ng DNA, 10 μM forward and reverse primers and 1×Syber green (CW0957M, Cwbio) on a LightCycler 96 Real-Time PCR System (Roche, Basel, Switzerland). The PCR program was as follows: 95 °C for 5 min, 40 cycles at 95 °C for 15 s, 60 °C for 15 s, 72 °C for 60 s. Differences were determined by 2-ΔΔCT analysis calculated using endogenous housekeeping gene (β-actin) and respective controls. The primers used are listed in Table S3.

LC-MS/MS Analysis. After treatment with FLAG-tagged CNF1 (3 nM) or dialysis buffer for 6 h, BMDMs cells were washed with pre-chilled PBS and lysed in lysis buffer (2 mM EDTA, 50 mM Tris-HCl pH 7.4, 150 mM NaCl, 1% NP-40, 20% glycerol) with complete mini-EDTA-free protease inhibitor cocktail tablets (11697498001, Roche). Cell supernatants were centrifuged to remove cell debris and incubated with anti-FLAG M2 beads (A2220, Sigma) for 12 h at 4 °C with constant rotation. After washing, anti-

FLAG M2 beads were then incubated with 3×FLAG Peptide (F4799, Sigma) to elute the FLAG protein complex. The elutes were collected and visualized on 10% NuPAGE Bis-Tris gel (NP0301BOX, Thermo Fisher) followed by protocol of silver staining kit (24612, Thermo Fisher). The differential bands between the dialysis buffer group and the FLAG-tagged CNF1 group was retrieved and analyzed by LC-MS/MS. Data were analyzed using a full-scan mass spectrum (300 to 1800 m/z). Finally, Proteome Discoverer (version 1.4.0.288, Thermo Scientific) was used to analyze data.

Immunofluorescence analysis. BMDMs and HeLa cells were pre-cultured in LAB-TEK 4-well chamber slides (177399, Thermo Scientific), vigorously washed with PBS, and fixed with 4% paraformaldehyde for 30 min. After washing, cells were permeabilized with 0.5% Triton X-100/PBS for 15 min, blocked with PBS containing 5% BSA for 1 h at room temperature, and stained with specific primary antibodies against FlAG (8146, CST), JAK1 (8146, CST), JAK2 (3230, CST), STAT1 (9172T, CST), pSTAT1 (Tyr701) (7649, CST), EEA1 (ab109110, Abcam, Cambridge, MA, USA), and LAMP1 (ab208943, Abcam) in blocking buffer overnight at 4 °C. After that, slides were incubated with appropriate Alexa Fluor 488/594-conjugated secondary antibody (SA00013-2 and SA00013-3, Proteintech) for 1 h and counterstained with DAPI. Images were acquired using a confocal fluorescence microscope (Leica TCS-SP8, Leica Microsystems). The intensity of fluorescence was quantified using the software Image Pro Plus (Media Cybernetics, Silver Spring, MD, USA). Quantification of colocalization was calculated by using Image Pro Plus software.

Immunohistochemistry. Kidneys from mice were fixed in a solution of 3% formalin in PBS for 24 hours and embedded in paraffin. Paraffin sections (5 μm) of tissues were deparaffinized, dehydrated, and then subjected to antigens retrieval in citrate buffer (pH 6.0) or Tris-EDTA buffer (pH 9.0).The sections were incubated with 0.3% hydrogen

peroxide for 15 min, blocked with 5% normal goat serum for 1 h, and stained overnight with anti-F4/80 (28463-1-AP, Proteintech, Chicago, IL, USA), anti-pSTAT1(Tyr701) (9167S, Cell Signaling Technology, Danvers, MA, USA), and anti-pIκBα (Ser32) (AF2002, Affinity Biosciences, Cincinnati, OH, USA).After washing with PBS, the sections were stained for 30 min with horseradish peroxidase (HRP)–labeled secondary antibody (PV-6001, Zsbio, Beijing, China) and then visualized with diaminobenzidine chromogenic substrate (ZLI-9019, Zsbio). Images were acquired with a microscope (BX46, Olympus, Tokyo, Japan). The intensity of staining was quantified using the software Image Pro Plus.

Antibodies and western blotting. Anti-β-actin (A1978) and anti-FLAG (F3165) antibodies were purchased from Sigma-Aldrich (St. Louis, MO, USA). Anti-JAK1 (3344S), anti-pJAK1 (Tyr1034/1035) (3311S), anti-JAK2 (3230T), anti-pJAK2 (Tyr1007) (4406T), anti-HA (3724s), anti-IκBα (9242S), anti-pIκBα (2859S), anti-pSTAT1 (7649T), anti-pSTAT3 (9145T), anti-pSTAT5 (4322T), anti-STAT1(9172T), anti-STAT3 (12640S) and anti-STAT5 (25656S) antibodies were purchased from Cell Signaling Technology (Danvers, MA, USA). Anti-MYC antibody (66004-I-Ig) was purchased from Proteintech (Chicago, IL, USA). Cells were lysed in RIPA lysis buffer (R0020, Solarbio) with addition of complete mini-EDTA-free protease inhibitor cocktail tablets (11697498001, Roche) and phosphatase inhibitor cocktail tablets (4906845001, Roche). After centrifugation, the protein supernatants were quantified by a BCA Protein Assay Kit (23225, Thermo Fisher) and boiled for 10 min in SDS loading buffer (CW0027S, ComWin). An equal amount of protein from all the samples was separated by SDS-PAGE and then transferred to PVDF membranes. The PVDF membrane was blocked with 5% milk or BSA and incubated with indicated primary antibodies and corresponding HRP-conjugated secondary antibodies. Signals were

detected by GE Amersham Imager 600 machine (GE Healthcare, Chicago, IL, USA). Densitometry was performed using ImageJ software (National Institutes of Health, Bethesda, USA).

Immunoprecipitation (IP) assays. HeLa or BMDM cells were washed with pre-chilled PBS and lysed in lysis buffer containing complete mini-EDTA-free protease inhibitor cocktail tablets on ice. Cell supernatants were incubated with anti-FLAG M2 beads (A2220, Sigma) or anti-c-MYC beads (A7470, Sigma) for 12 h at 4 °C with constant rotation. After washing, beads were boiled in SDS loading buffer and subjected to SDS-PAGE and western blotting.

For endogenous JAK1 protein IP, cell supernatants were incubated with anti-JAK1 antibody (50996S, CST) overnight at 4 °C, and then incubated with protein A/G agarose (20241, Thermo Fisher) for 2 h at 4 °C with constant rotation. Normal rabbit IgG (2729S, CST) was used as the control. After incubation, beads were thoroughly washed with lysis buffer, boiled in SDS loading buffer and subjected to SDS-PAGE and western blotting.

For immunoprecipitation in vitro, purified recombinant FLAG-tagged protein (1 μg) was incubated with recombinant MYC-tagged protein (1 μg) or recombinant GST-tagged protein (1 μg) in binding buffer (20 mM Tris-HCl pH 7.4, 0.1 % Triton-X 100, 100 mM NaCl, 20% glycerin, 1% BSA) for 12 h. Mixture was subsequently incubated with anti-c-MYC beads (A7470, Sigma) for 12 h at 4 °C with constant rotation. After washing, beads were boiled in SDS loading buffer and subjected to SDS-PAGE and western blotting.

In vitro kinase assay. Fusion proteins (FLAG-tagged CNF1, FLAG-tagged C866S, FLAG-tagged N1, FLAG-tagged N2 were incubated with recombinant MYC-tagged JAK1 and MYC-tagged JAK2 protein for 30 min at 30 °C in kinase buffer (9802S, CST)

containing 500 μM ATP (9804S, CST). The reactions mixture was boiled in SDS loading buffer and subjected to western blotting.

In vitro droplet assay. Recombinant mCherry fusion proteins or recombinant GFP fusion proteins were concentrated at indicated varying concentrations and added to solutions with indicated final salt and 10% PEG-8000 (polyethylene glycol, molecular weight 8000) (P5413, sigma). The protein solution was loaded onto a glass slide with a coverslip. Slides were then imaged with a confocal fluorescence microscope (Leica TCS-SP8, Leica Microsystems).

FRAP analysis. Formed droplets were bleached with ZEISS LSM 800 (ZEISS, Oberkochen, Germany) using a 594-nM laser of a confocal microscope with a 63×/1.4 oil objective. Recovery was imaged on ZEISS LSM 800 confocal laser scanning microscope every 15 s for the indicated time periods.

Fluorescent imaging for LLPS in vivo. HeLa cells were treated with mCherry-CNF1, mCherry-N2 and mCherry proteins (15 nM) for 6 h and washed with PBS three times. Cells images were acquired on a Leica TCS SP8 microscope.

1, 6-Hexanediol treatment. HeLa cells were treated with mCherry-CNF1, mCherry-N2, and mCherry (15 nM) for 6 h. After washing, cells were imaged. For BMDMs, cells were treated with FLAG-tagged CNF1/FLAG-tagged N2 (3 nM) or dialysis buffer for 6 h. After immunofluorescent staining, BMDMs were imaged. For HeLa cells overexpressing JAK1-GFP and JAK2-mCherry, CNF1/N2 (15 nM) or dialysis buffer was used to treat for 6 h. Following the first acquisition, 3% 1, 6-hexanediol (H11807, Sigma) was added to the plate to acquire the second image at different times.

Clodronate liposomes and inhibitors treatment in vivo. To eliminate macrophages, PBS or clodronate liposomes (CP-005-005, Target Technology, Beijing, China) were administered to mice intravenously 24 h before infection. For NF-κB inhibition and

STAT1 inhibition, single dose of 20 mg/kg Bay 11-7085 (HY-10257, MedChem Express) or 40 mg/kg Fludarabine (HY-10257, MedChem Express) dissolved in DMSO was intraperitoneally injected each day for 3 days before infection, respectively. Mice were euthanized at 12 hpi or 24 hpi. Kidneys were collected for flow cytometry and renal histopathology analysis.

H&E staining and assessment of kidney pathological score. Kidneys from mice were fixed in a solution of 3% formalin in PBS for 24 hours and embedded in paraffin. Paraffin sections (5 μm) of tissues were stained with hematoxylin and eosin (H&E). Images were captured with a microscope (BX46, Olympus, Tokyo, Japan). Renal histopathological changes was graded using a 6-point scale according to tissue destruction, cellular infiltration, bacterial patchiness and presence of abscesses, in which 0, 1, 2, and 3 indicated normal, mild, moderate, and severe pyelonephritis, respectively (pathological changes were mainly located within the cortical-medullar junction and the medulla); while 4, 5, and 6 indicated mild, moderate, and severe pyelonephritis (pathological changes was located in more parts of the kidney), respectively. The assessment of kidney pathological score was performed by two persons who were blinded to experimental groups.
